# Infant limb kinematics and muscle force variation during kicking is associated with clinical cerebral palsy risk measures

**DOI:** 10.1371/journal.pone.0357214

**Published:** 2026-09-08

**Authors:** Nidal S. Khatib, Luca J. Schmidtke, Anna Lukens, Tomoki Arichi, Silvia Muceli, Etienne Burdet, Bernhard Kainz, Niamh C. Nowlan

**Affiliations:** 1 School of Engineering and Materials Science, Queen Mary University of London, London, United Kingdom; 2 School of Mechanical and Materials Engineering, University College Dublin, Dublin, Ireland; 3 Department of Computing, Imperial College London, London, United Kingdom; 4 Department of Bioengineering, Imperial College London, London, United Kingdom; 5 Dept. AIBE, Friedrich-Alexander-Universität Erlangen-Nürnberg, Erlangen, Germany; 6 Neonatal Unit, Evelina London Children’s Hospital, Guy’s and St Thomas’ NHS Trust, London, United Kingdom; 7 Early Life Imaging Research Department, School of Biomedical Engineering and Imaging Sciences, King's College London, London, United Kingdom; 8 MRC Centre for Neurodevelopmental Disorders, King’s College London, London, United Kingdom; 9 Guy’s and St Thomas’ NHS Foundation Trust, King’s College London, London, United Kingdom; 10 Chalmers University of Technology, Goteborg, Sweden; Drexel University School of Biomedical Engineering Science and Health Systems, UNITED STATES OF AMERICA

## Abstract

Early limb motor development progresses from spontaneous to increasingly complex movements. Restricted movement variety in infancy can indicate neuromotor impairment associated with cerebral palsy (CP) risk. The General Movement Optimality Score (GMOS) used in clinic detects abnormal movement patterns, but requires highly trained physiotherapists, limiting accessibility. Objective biomechanical measures may provide quantitative predictors of CP risk, reducing clinical burden while offering novel physiotherapeutic targets. This study investigated whether variation in lower limb kinematics and muscle forces during spontaneous kicking is reduced in infants with lower GMOS scores, indicating higher CP risk. We also examined whether variation correlates with gestational and corrected age, reflecting the transition to more complex movements. Twenty-six Infants aged 0–3 months underwent wired electromagnetic motion capture (Polhemus Liberty System) with musculoskeletal modelling to estimate kinematics and muscle force waveforms, alongside GMOS scoring. Waveform variation was quantified using functional principal component analysis and correlated with GMOS scores and age, while partial least squares discriminant analysis (PLS-DA) identified biomechanical predictors of GMOS indications of ‘normal’ versus ‘poor repertoire’ (PR) movements. Variation in knee flexion, hip flexion and abduction kinematics, and rectus femoris, pectineus, biceps femoris and gemelli forces significantly correlated with GMOS scores. Gestational age correlated with hip abduction, ankle dorsiflexion, and gemelli and medial gastrocnemius forces. PLS-DA achieved 85% accuracy distinguishing normal from poor repertoire movements, with knee and hip kinematics and biceps femoris force as key discriminators. These findings demonstrate that lower limb kinematic and neuromuscular variation aligns with clinical motor assessments, supporting their potential as objective biomechanical markers of CP risk.

## Introduction

Cerebral palsy (CP) is a motor impairment characterised by irregular movement, coordination, and muscle tone resulting from abnormal brain development or birth-related brain damage [1,2]. CP is more common in babies born preterm, with a prevalence of 9.1% in pre-term births, up to around 40% in extremely preterm births, compared to just 0.1% for those born at term [3,4]. Diagnosis of CP early in life is critical to improving the long-term outcomes of the disorder and to optimising infant motor and cognitive plasticity, but early diagnosis is challenging [1,5,6]. The diagnostic process in the first few months of life is resource-intensive, and requires the collaboration of several highly trained clinicians, including paediatricians, neurologists, and physiotherapists, to perform a series of clinical observations, movement assessments, and magnetic resonance imaging (MRI). The development of novel objective and accurate methods to quantify and predict abnormal general movements could support early diagnosis of CP and help to reduce the costs associated with clinician training and time.

Abnormal general movements detected during the first five months of age can be strongly indicative of cerebral palsy. Prechtl’s General Movement Assessment (GMA) is a clinical evaluation of general movements that most reliably diagnoses CP, with a sensitivity of 98% (95% CI: 74–100%) and specificity of 91% (95% CI: 83–93%) for infants in the fidgety stage (three to five months of age), however accuracy is reduced in earlier stages [5,7]. In the assessment, infants are categorised according to their quality of movement, which is used to inform diagnosis and potential intervention. The variety and quality of movements are scored considering parameters of Gestalt perception, which considers the variety of movement sequence, range of motion, speed, onset and offset, and intensity [7]. The general movement optimality score (GMOS) is a scoring system based on the GMA, which adds a semi-quantitative component [8]. Limited variety of movement, given a lower score in the GMOS system, can be a sign of neurological issues and is associated with a higher risk of CP development. Due to the semi-quantitative approach to identifying abnormal general movements, the GMOS provides an opportunity to develop objective biomechanical predictors of CP risk in line with clinical measures.

A major challenge of applying the GMA or GMOS in practice is the cost and availability of appropriately skilled clinicians, particularly in cases where there is a requirement for multiple observers or opinions, or increased susceptibility to inter-observer variability or observer fatigue. Developing objective tools could enable automated diagnoses of abnormal general movements. Such tools would be valuable when specialist clinicians are scarce, when verifying GMA results, during regular re-testing post-intervention, or for training purposes. There has been a recent growing interest in computer-based approaches for detecting general movements with more sophisticated tools emerging yearly [9,10]. Notably, attempts have been made to analyse general movements from videos using computer vision approaches such as automatic movement recognition using video data or pressure mats combined with machine learning or artificial intelligence [9–17], and biomechanical analysis from motion capture or IMU data [10,18–21]. The results from many of these studies are promising, showing detection of abnormal movements with sensitivities between 73–90% or classification accuracies of 78–88%. Previous work in this area has largely focussed on classifying infants into binary categories such as ‘abnormal’ versus ‘normal’, or ‘fidgety’ versus ‘non-fidgety’ movements. Given the semi-quantitative nature of identifying abnormal general movements, generating predictors for the GMOS score allows for the creation of continuous-scale predictors, potentially better suited for determining impairment severity or individual developmental trajectories [22].

Previous studies on the normal maturation of infants’ limb kinematic movements have highlighted the transition from synchronised hip, knee and ankle movement coordination to less in-phase, decoupled and varied movement patterns [23–31]. Kinematics analyses have also been used to predict lateral movement disorders due to brain white matter damage (WMD). Preterm or term infants at the same age with WMD lesions display lower correlations and higher standard deviations of hip-ankle and knee-ankle paired movements than preterm infants without WMD, indicating that WMD leads to more constrained movements [23,31]. In addition, WMD-affected infants experience reduced spatiotemporal parameter variability during kicks compared to control infants of the same age range [31]. While previous work has correlated a change in kinematics with infantile brain development and damage, we are not aware of prior attempts to associate parameters such as variability of movement with GMOS scores, which are particularly sensitive to CP detection.

Since the GMOS aims to capture variation of movement sequence, amplitude, speed and intensity of general movements, such as upper and lower limbs, motion capture combined with 3D musculoskeletal modelling is well suited to the identification of representative biomechanical markers. Musculoskeletal modelling can represent a wider array of movements in three-degree-of-freedom joints, including hip flexion-extension, abduction-adduction, and internal-external rotation, capturing a greater range of movement variations within joints compared to traditional kinematic modelling. Furthermore, musculoskeletal modelling can estimate the variation in muscle activation forces, which sometimes influence multiple joints at once. Modelling muscle forces can enhance our understanding of neuromuscular deficits in infants with neuromotor impairments. Such insights can improve physiotherapy treatments and assist in monitoring neuromotor function over time. Functional principal component analysis (fPCA) is an extension of PCA which reduces and examines the variation in human movement waveform data [32,33]. When paired with musculoskeletal modelling, fPCA offers a deeper analysis of biomechanical waveforms compared to discrete parameterisation often employed in biomechanics studies, because it considers both the spatial and temporal changes in the entire biomechanical waveform. Its output measures, known as PC scores, might be a better representation of the overall variation in general infant movements and could lead to stronger predictors of GMOS scores compared to individual parameters. As fully automated markerless 3D pose estimation and musculoskeletal modelling workflows emerge [13,15,34,35], identifying biomechanical predictors for abnormal movement could be valuable for developing cost efficient automated diagnostic tools that additionally provide physiological indicators of disease, underscoring the timeliness of this work.

In this study, we investigate the potential of combining motion capture of the infantile lower limbs with 3D musculoskeletal modelling and functional PCA for prediction of the GMOS score. We first report the normal ranges of lower limb kinematic and muscle forces during kicking movements. We then test the hypothesis that variation in lower limb kinematic and muscle force outputs during common kicking movements is reduced in infants with lower GMOS scores, which are at a higher risk of CP development. In addition, we test the hypothesis that variation in kinematic and muscle force outputs are positively related to gestational age at birth and corrected age. Finally, we carry out a partial least squares discriminant analysis (PLS-DA) to determine whether it is possible to discriminate infants categorised as ‘normal’ or ‘poor repertoire’ of general movements in the GMOS using objective kinematic and muscle force waveform parameters, and identify the strongest biomechanical predictors of the GMOS score.

## Methods

### Subject recruitment

Approval for the collection of experimental data from 45 preterm or full-term infants was obtained (IRAS 263765, REC: 19/LO/1384 and IRAS 257568, REC: 12/LO/1247). Written parental consent was obtained prior to data collection. Data collection took place from 1st January 2019–1st January 2020. Retrospective medical records were collected on the 15^th^ September 2021. Preterm infants were included if born at <37 weeks gestation, and excluded if there was a known diagnosis of a genetic or chromosomal anomaly or metabolic condition. Full term infants were eligible for inclusion if born between 37–42 weeks gestation and had been assessed to be clinically stable for study by a specialist physiotherapist and/or paediatrician (A.L./T.A.). We recruited the largest feasible sample within the ethical and logistical constraints of our clinical setting and available patient cohort.

### Clinical assessment of general movements

The GMOS was carried out by a specialised neonatal physiotherapist (author A.L.) in person to evaluate the general movements of infants (Supplementary materials). Neonatal infants were placed in the supine position in a cot and allowed to move freely for a period of 7 minutes during the assessment. The GMOS assessment has two independent parts: the global assessment and detailed scoring. The global assessment differentiates between the categories of ‘normal’, ‘poor repertoire’ (PR), ‘cramped-synchronised’ and ‘chaotic’ general movements [8]. A ‘normal’ category is assigned if the movements are variable, whereas a ‘PR’ is given when movements are monotonous and lack the normal variability. Cramped-synchronised and chaotic infants were excluded due to low numbers. The detailed scoring considers the sequence, amplitude, speed, spatial range, proximal and distal rotations, onset and offset, tremulous and cramped components of the movements. Each of the categories is scored between 0–2, for a total summed GMOS score out of 42, whereby a higher score indicates normal, variable, and smooth movements and a lower score indicates monotonous, jerky and cramped movements. Einspieler et al. [8], when examining 233 infants determined that infants assigned the ‘normal’ category scored between 30–42, while infants in the ‘PR’ category scored between 13–39.

### Motion capture and musculoskeletal modelling

**Fig 1:**
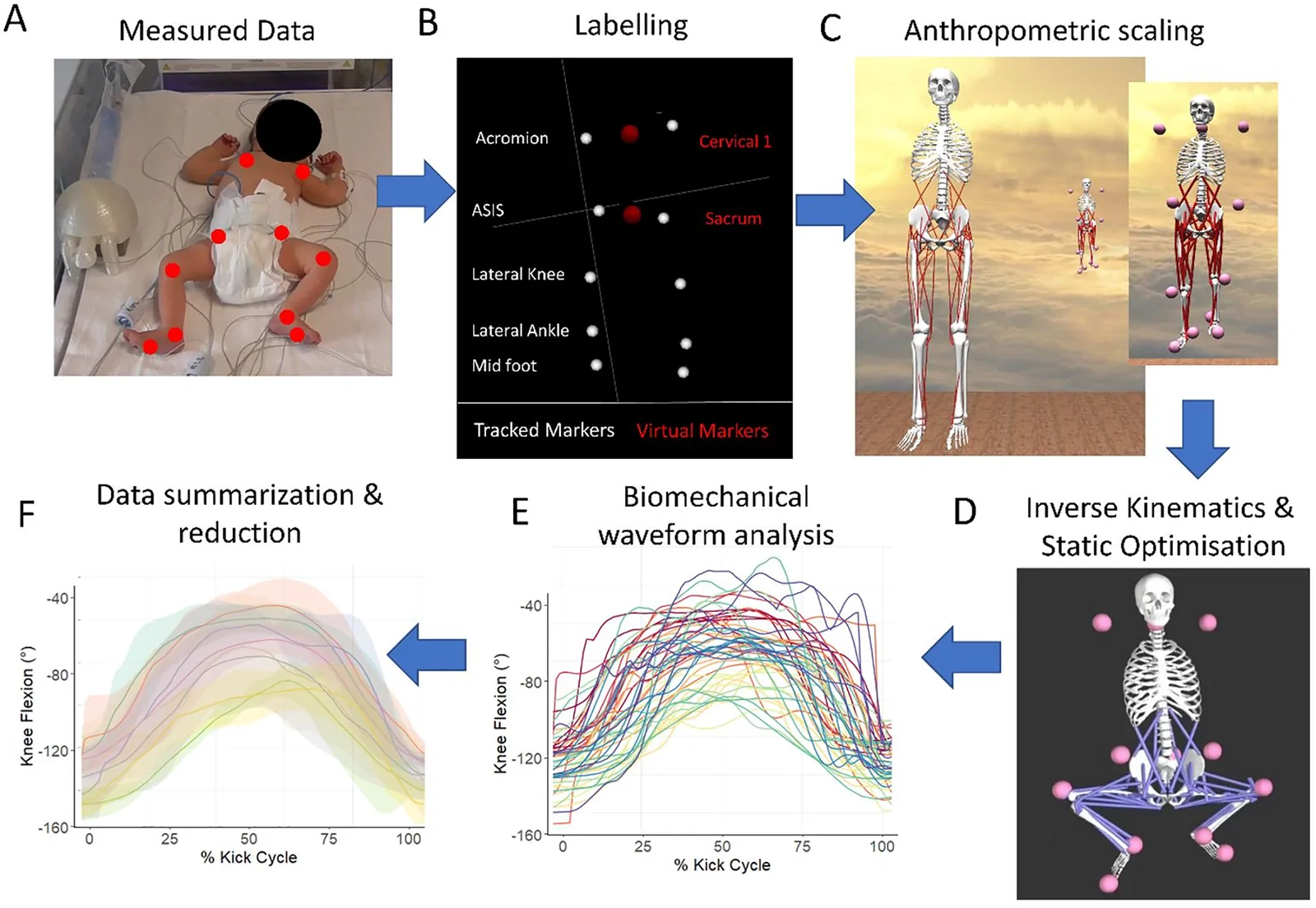
Musculoskeletal modelling pipeline. (A) Electromagnetic motion capture data collected at 120 Hz from 10 sensors attached to anatomical landmarks (Polhemus Liberty, Polhemus, US). (B) 3D coordinates were Butterworth filtered with 6 Hz cut-off. Additional virtual markers were generated to scale and orient the torso and pelvis segments. (C) Experimental and virtual markers placed on GAIT2354 OpenSim model to scale to subject proportions and orient segments to a common static pose. (D) Inverse kinematics and static optimisation carried out frame by frame to estimate lower limb kinematics from 3D coordinate data and estimate muscle forces for 5-minute movement sequences. (E) Biomechanical waveforms of kicking cycles extracted from movement sequences and time normalised. (F) Means and standard deviations calculated to display normative data, and data reduction techniques (functional principal component analysis) carried out for comparative analysis.

Motion capture was carried out at the same time as the GMOS assessment, using an electromagnetic motion capture system (Polhemus Liberty, Polhemus, US). Wired electromagnetic sensors were attached to the infants using hypoallergenic tape to ten anatomical landmarks, five on each side of the body on the acromion, anterior superior iliac spine (ASIS), lateral knee, lateral ankle and midfoot inferior (underside of the foot) as shown in Figs 1A and 1B. 3D coordinate data was collected at 120 Hz for a total of five minutes (Fig 1A). Captured 3D sensor coordinates were filtered using a fifth order Butterworth filter with a cut off frequency at 6 Hz in R and exported to OpenSim for musculoskeletal modelling.

Musculoskeletal models of the lower limbs and torso were generated in OpenSim 4.3 [36]. The Gait2354_simbody model was selected due to its specific development for lower limb modelling. The knee and ankle joints were modelled as hinge joints (flexion-extension), and the hip joints as ball and socket joints (flexion-extension, adduction-abduction, internal rotation-external rotation). Model segments (torso, pelvis, femur, tibia, and foot) were scaled to the proportions of each subject, using marker positions obtained from a static calibration snapshot in the supine position with knees fully flexed and hips extended (Fig 1C). Bodyweight of the model was based on bodyweight of the infants acquired in clinic at the time of assessment. Inverse kinematics was applied to the scaled subject models to generate kinematic waveforms of the whole movement sequence for hip flexion, abduction and rotation, knee flexion, and ankle dorsiflexion (OpenSim). Static optimisation was then carried out using inverse kinematics outputs, to generate estimated muscle force waveforms for 24 lower limb muscles.

To assess the kinematics and muscle force during kicking movements, kicking sequences were identified from the filtered knee flexion data output. Knee flexion data was used to identify the kicking movements due to the clear onset and offset of the kicking cycles in the data. A custom R script was used to label time stamps for segments of the knee flexion waveforms that included an initial knee extension of at least 40° with a subsequent knee flexion of at least 40° within a 2-second time period (RStudio). This threshold was based on a combination of previous studies examining kicking movements in infants under 6 months of age [21,23,24,26]. Manual checking and minor adjustments ensured the full kick movements were captured and the exclusion of output errors. At least 4 kicks from each individual infant were required in order to be included for analysis, and only a single limb was used per infant to avoid pseudoreplication and to ensure statistical independence, as GMOS scores are assigned at the whole-infant level. Given the generally symmetric nature of spontaneous kicking behaviour in early infancy, one limb was considered representative of overall movement patterns and was selected based on the number of recorded kicks and overall data quality when both limbs were available. Time intervals for labelled kicking sequences were used to crop all kinematic and muscle force waveforms to isolated kicking measurements using a custom R script (R studio).

### Functional principal component analysis

**Fig 2:**
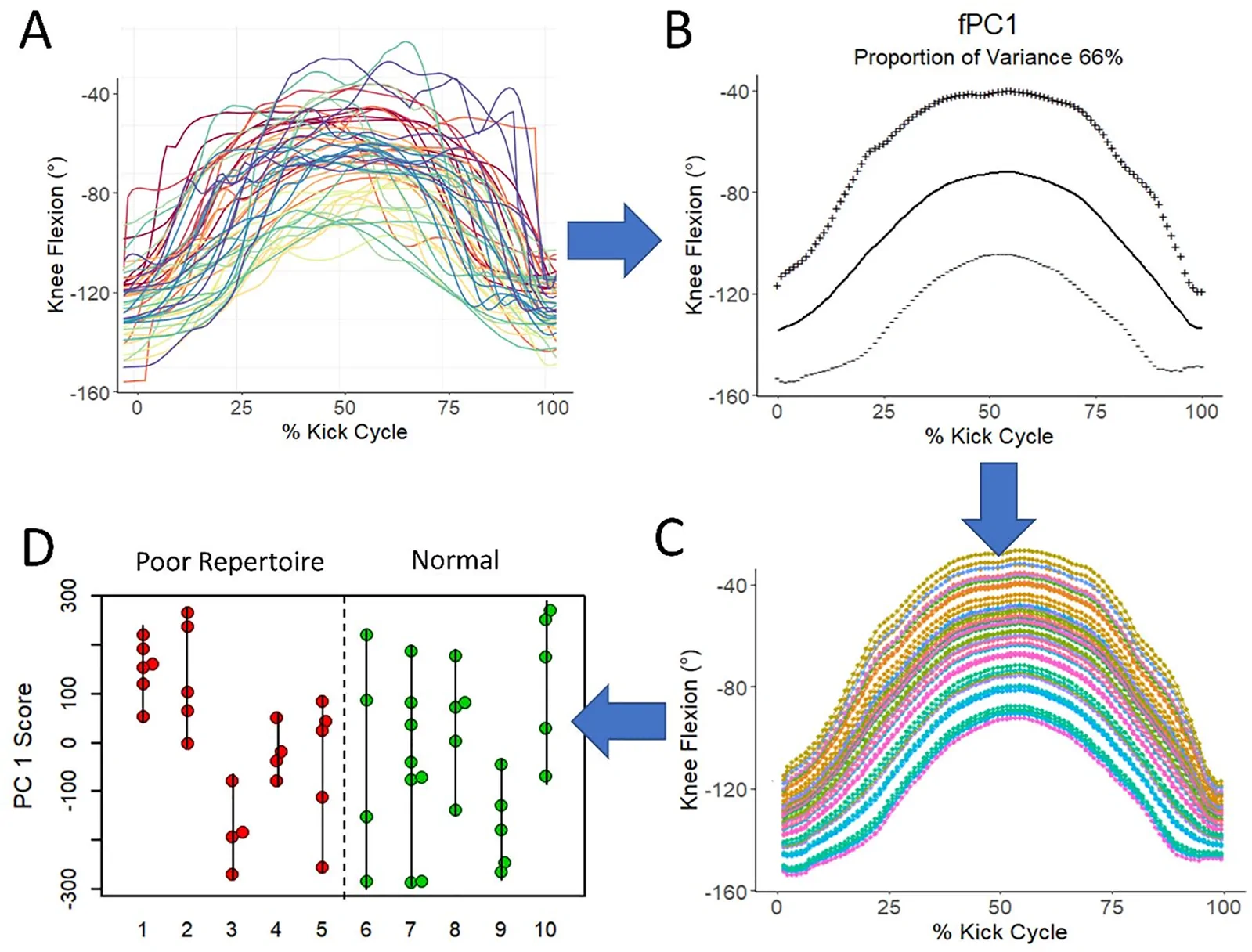
Functional principal component analysis (fPCA) of biomechanical waveforms. (A) Biomechanical waveform data from knee and hip kinematics and muscle forces were time normalised. (B) Functional PCA was carried out on biomechanical waveforms (*X* *=* 101 points of the kick cycle) using the mathematical method previously described [32] (C) Reconstruction of the original waveform data from the individually calculated principal component (PC) loading vectors. Reconstructions were used to interpret the feature of variance captured by the PCs. (D) PC scores (one score per kick) for individual subjects were calculated and the variances in scores (standard deviations) were correlated against the General Movement Optimality Scores (GMOS), gestational age at birth and corrected age at assessment.

fPCA was employed to quantify the intra- and inter-subject variation in kinematic and muscle force waveforms during kicking movements. fPCA was chosen for variance quantification as it considers both spatial and temporal variation over the entire biomechanical waveform, as opposed to discrete parameterisation which considers peak and range values, thus providing greater sensitivity than discrete parameter analysis. Waveforms cropped to kicking intervals were resampled to 101 points for standardisation and data compression. fPCA was applied to transform waveforms into “principal modes of variation”, which capture the patterns of highest variation across the whole curve [32](Fig 2). For each parameter, the first principal component (PC) capturing the most common pattern of variation of the waveform was added, followed by the second, and so on, until at least 85% of the variation in the original waveform data was captured. Then, PC scores for each kick of each infant were calculated, indicating how much each waveform aligns with each mode of variation (Fig 2). The standard deviations (SDs) of the PC scores for each subject were then calculated to represent the amount of intra-infant variation for each mode of movement variation captured. Finally, to interpret the specific patterns of variation captured by the PCs, we reconstructed the original waveforms from the PCs, following the method previously described [33].

### Statistical analysis

Pearson’s correlations were carried out to determine firstly the relationship between the SDs of PC scores for each infant (biomechanical variation) and the GMOS scores, gestational age at birth, and the corrected age at time of assessment. Values of significance were Bonferroni corrected to account for multiple testing.

### Partial least squares – discriminant analysis

A partial least squares discriminant analysis (PLS-DA) model was constructed with two latent variables (components) to discriminate between infants categorised as ‘normal’ and ‘PR’ in the GMOS. The dataset included all 26 subjects, and predictor variables included [1] Clinical features including ‘corrected Age’ and ‘gestational Age,’ and [2] SDs of PC scores related to lower limb kinematics and muscle force curves that significantly correlated with the GMOS score. Missing values were imputed using the mean imputation method in 3 cases where abnormal hip, knee or ankle measurements for particular subjects were removed due to errors in the musculoskeletal model. All continuous features were Z-score standardised. The model was validated, and accuracy assessed using leave-one-out cross validation. Sensitivity (true positive rate) and specificity (true negative rate) were calculated by comparing predicted classifications to the GMOS-based group labels (‘poor repertoire’ and ‘normal’). Sensitivity was defined as the proportion of correctly identified ‘poor repertoire’ infants, while specificity was defined as the proportion of correctly identified ‘normal’ infants. Permutation testing with 1000 iterations was employed to assess the model's statistical significance. A 2D plot was generated to visualise the separation between the groups in the reduced feature space, with a decision boundary computed using logistic regression to demarcate the classification regions.

## Results

### Subjects

Data was collected from a total of 45 infants. Following removal of subjects with limited kicking cycles and subjects in which marker data was unusable due to detachment of the sensors during the assessment, the final number of subjects was 26 infants. The subject population included 13 male and 13 female infants, of which 15 were born preterm (<37 weeks) and 11 at term. The average gestational age at birth was 34.9 (± 5.2) weeks. The timing of which the assessment was conducted was at an average of 4.8 (± 4.7) weeks of corrected age. GMOS scores ranged between 14 and 40, with an average score of 30.1 (± 6.0). This included 10 infants with a ‘normal’ outcome and 16 infants with a ‘PR’ of movement outcome. The ‘normal’ group scored an average of 36 (± 2.8) on the GMOS, while the ‘PR’ group scored on average of 26.4 (± 4.3). While there was a significant difference in GMOS scores between the ‘normal’ and ‘PR’ groups (p < 0.001), there were no significant differences in GMOS scores relative to the gestational age at birth or corrected age at assessment.

### Normative ranges of kinematics and muscle forces during kicking

**Fig 3:**
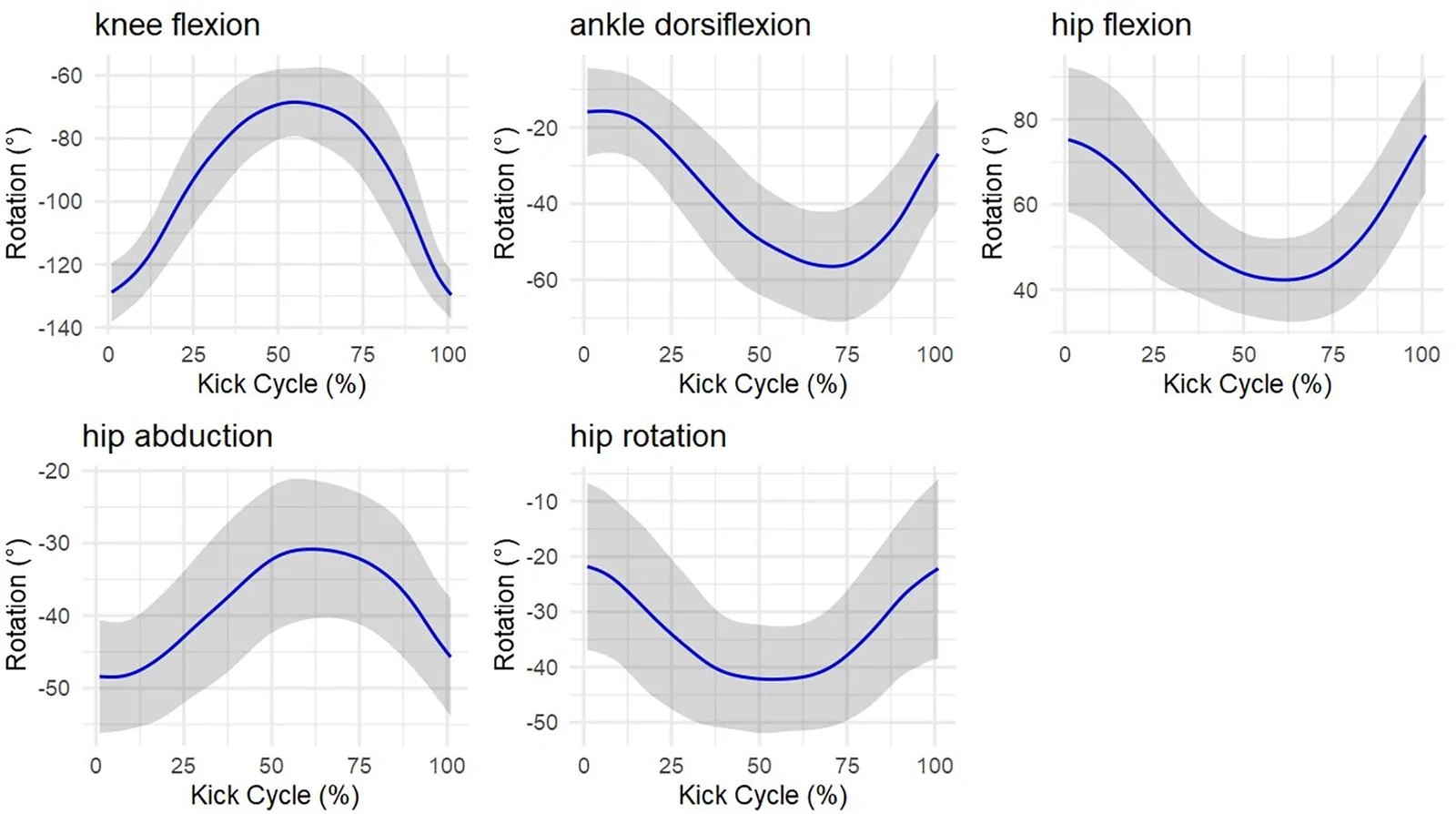
Ensemble averaged knee, ankle and hip kinematic curves for a full kick cycle, representing all subjects (n = 26). A single kicking cycle represents knee flexion to knee flexion. Blue lines; mean curves, shaded areas; standard deviations.

**Fig 4:**
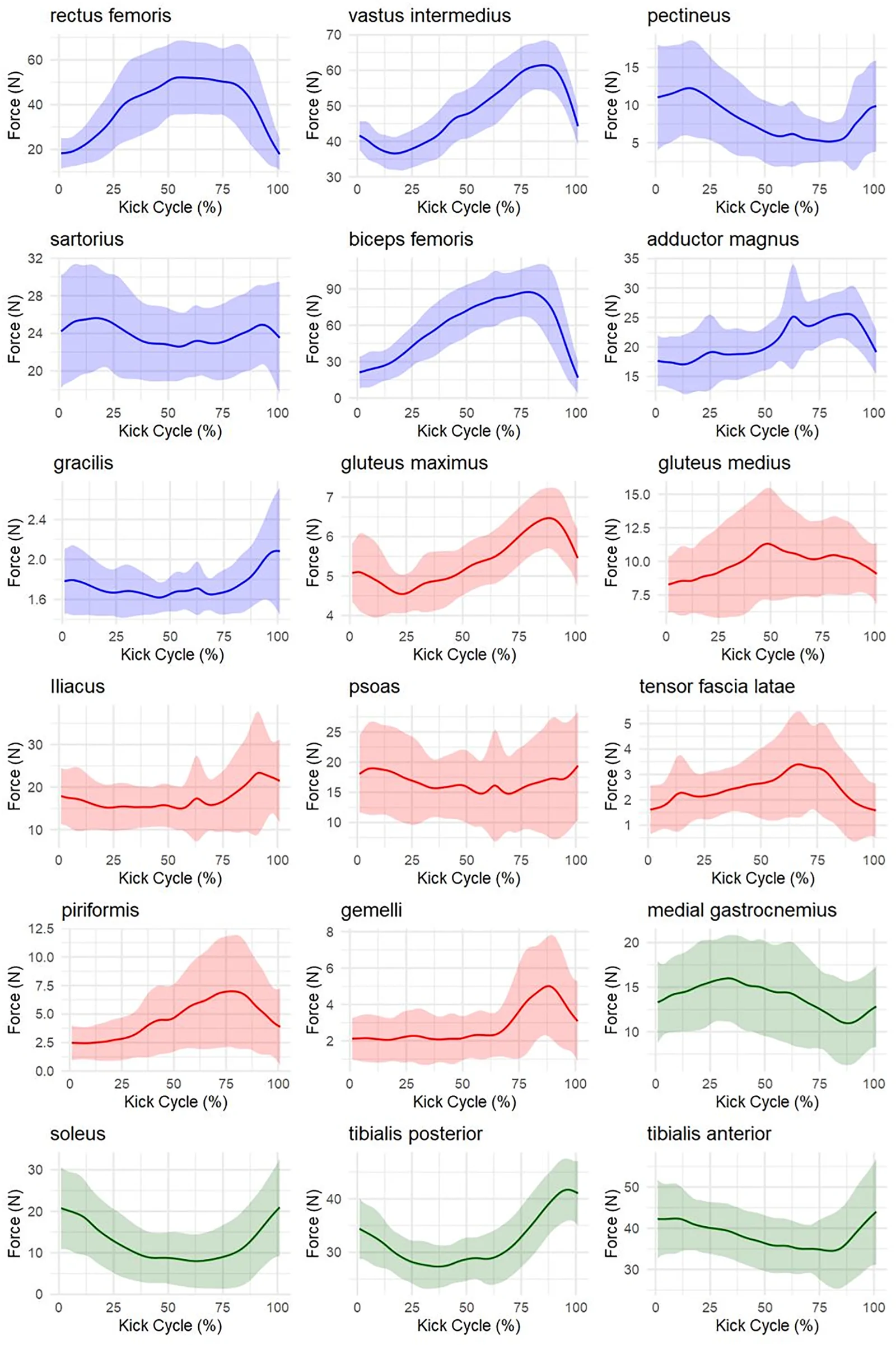
Ensemble averaged upper leg (blue), hip (red) and lower leg (green) muscle force curves for a full kick cycle, representing all subjects (n = 26). A single kicking cycle represents knee flexion to knee flexion. Lines; mean muscle force curves, shaded areas; standard deviations.

A total of 109 kick cycles were isolated from a single leg of 26 infants (4–6 kicks per infant). Normative ranges of lower limb kinematics are reported in Fig 3. Normative ranges of lower limb muscle forces are shown in Fig 4. At the thigh, the rectus femoris, vastus intermedius and biceps femoris displayed the highest force outputs relative to all lower limb muscles, with the rectus femoris displaying a ramp in force in line with the degree of knee extension peaking mid-way through the kick cycle (50–60%), while the vastus intermedius and biceps femoris displayed a late-stage peak activation (75–90%). The pectineus, sartorius and adductor magnus displayed relatively lower forces during kicking but a consistent pattern of early (10%) and late (90–100%) activation peaks. At the hip, the gluteus maximus, piriformis, tensor fascia latae and gemelli showed a consistent late-stage kick peak in force, at around 75–90% of the kick cycle. In contrast, the gluteus medius, iliacus and psoas displayed high variation and minimal activation change throughout the kicking cycle. At the calf, the medial gastrocnemius showed a peak force around mid-way (35–50%) through the kick cycle, while the soleus, tibialis posterior and anterior showed consistent force patterns of an early- (0–10%) and late-stage (90–100%) peak activation.

### Variations in lower limb kinematic and muscle forces during kicking are associated with GMOS scores

**Fig 5:**
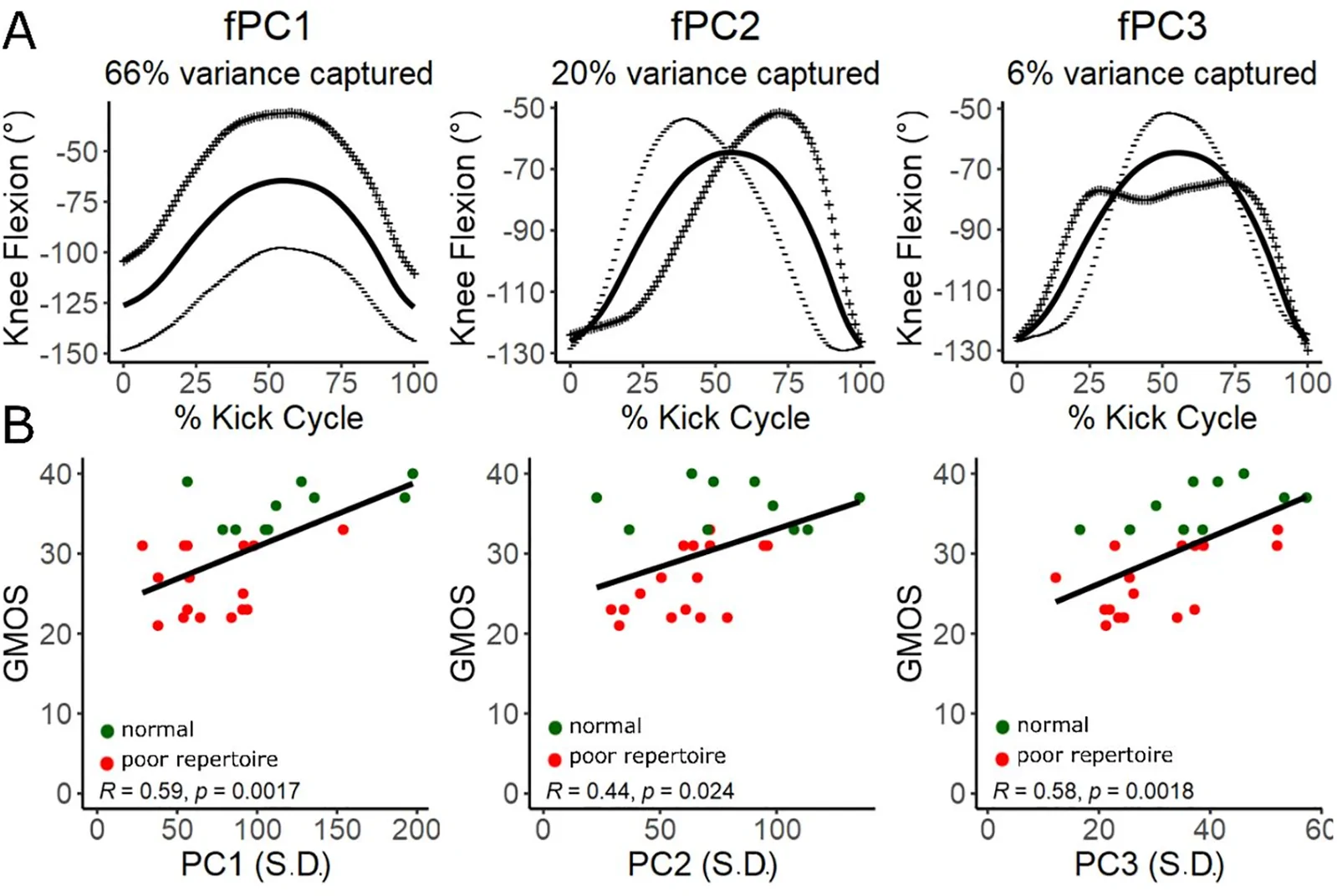
Variation in knee flexion during kicking is significantly associated (p < 0.05) with GMOS scores. A) Principal component (PC) reconstructions of the knee flexion curve. PC1: overall knee flexion magnitude, PC2: early-to-late kick knee extension, PC3: continual to bi-phasic kicking pattern. B) Pearson’s correlations of the standard deviation (SD) of PC scores for each subject and the GMOS scores. Solid line; mean. + line; mean +2 × SD. – line; mean −2 × SD. Red points represent infants assigned a ‘poor repertoire’ of movement score in the assessment. Green points represent infants assigned a ‘normal’ score. PC; principal component. SD; standard deviation. GMOS; general movement optimality score. Lower GMOS score = higher risk of CP. Variance captured; the variation of the original raw data captured by the PC.

**Fig 6:**
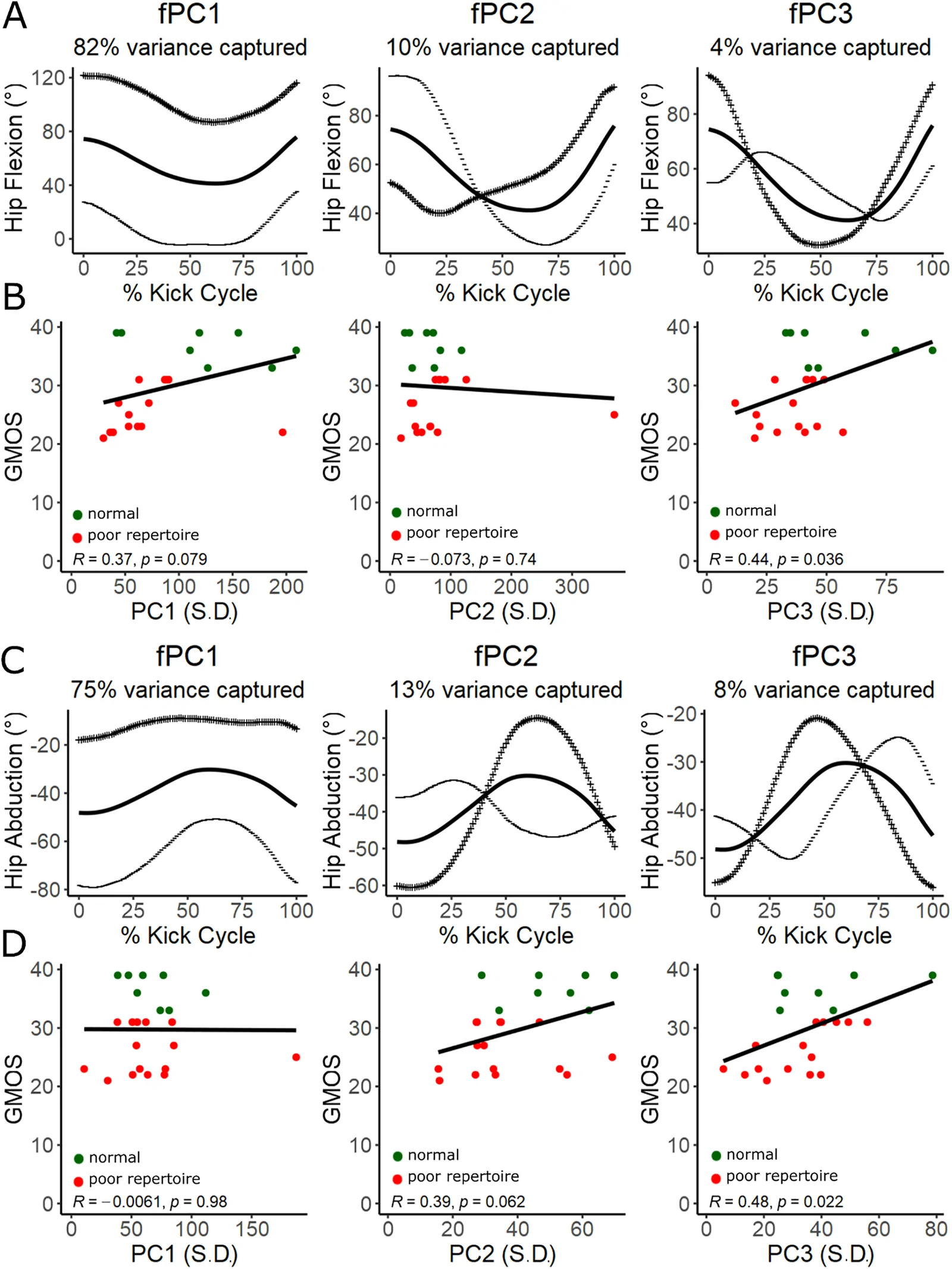
Variation in hip flexion and abduction during kicking is significantly associated (p < 0.05) with GMOS scores. A) PC reconstructions of the hip flexion curve. PC1: overall hip flexion magnitude, PC2: early-to-late kick hip extension, PC3: continual to bi-phasic hip flexion pattern. B) Pearson’s correlations of the standard deviation (SD) of hip flexion PC scores for each subject and the GMOS scores. C) PC reconstructions of the hip abduction curve. PC1: overall hip abduction magnitude, PC2: early-to-late kick hip abduction pattern, PC3: continual to bi-phasic hip abduction pattern. D) Pearson’s correlations of the standard deviation (SD) of hip abduction PC scores for each subject and the GMOS scores. Solid line; mean. + line; mean +2 × SD. – line; mean −2 × SD. Red points represent infants assigned a ‘poor repertoire’ of movement score in the assessment. Green points represent infants assigned a ‘normal’ score. PC; principal component. SD; standard deviation. GMOS; general movement optimality score. Lower GMOS score = higher risk of CP. Variance captured; the variation of the original raw data captured by the PC.

The variation (SD of PC scores) of the overall knee flexion magnitude (PC1, R = 0.59, p < 0.01), early-to-late kick knee extension peak (PC2, R = 0.44, p < 0.05), and single- to double-peak kicking pattern significantly and positively correlated with GMOS scores (PC3, R = 0.58, p < 0.01) (Table 1, Fig 5). At the hip, variation of the mid- to late-kick hip flexion (PC3, R = 0.44, p < 0.05), and the mid- to late-kick hip abduction was significantly associated with GMOS scores (PC3, R = 0.48, p < 0.05) (Table 1, Fig 6). While not significant, there were also moderate positive trends of variation of the overall hip flexion magnitude (PC1, R = 0.37, p = 0.079), hip abduction range of motion (PC2, R = 0.39, p = 0.062) and hip rotation range of motion (PC3, R = 0.41, p = 0.052) and GMOS scores (Table 1). Finally, while not significant, there were moderate trends found between variations in the ankle dorsiflexion range of motion (PC1, 68%, R = 0.38, p = 0.075) and early-to-late kick dorsiflexion peak with GMOS scores (PC2, 19%, R = 0.38, p = 0.071) (Table 1). Overall, our results indicate that variation in knee, hip and ankle movement during kicking are associated with GMOS scores.

**Table 1:** Statistically significant (p < 0.05) Pearson’s correlations between lower limb kinematic principal component standard deviations and the General Movement Optimality Score (GMOS), gestational age at birth and corrected age at assessment.

		GMOS		Gestational Age (at birth)		Corrected Age (at assessment)	
		R	*p*	R	*p*	R	*p*
Knee flexion	PC1	**0.59**	**0.0017**	−0.12	0.55	−0.25	0.21
	PC2	**0.44**	**0.024**	0.06	0.77	0.019	0.93
	PC3	**0.58**	**0.0018**	0.31	0.12	0.16	0.43
Ankle flexion	PC1	0.38	0.075	0.27	0.22	0.11	0.6
	PC2	0.38	0.071	**0.53**	**0.0098**	0.2	0.37
	PC3	0.3	0.16	**0.52**	**0.011**	0.3	0.16
Hip flexion	PC1	0.37	0.079	−0.044	0.84	−0.17	0.43
	PC2	−0.073	0.74	0.015	0.94	−0.14	0.53
	PC3	**0.44**	**0.036**	−0.3	0.16	−0.077	0.73
Hip abduction	PC1	−0.0061	0.98	0.037	0.87	−0.28	0.19
	PC2	0.39	0.062	0.056	0.8	−0.14	0.51
	PC3	**0.48**	**0.022**	**0.49**	**0.017**	**0.4**	**0.061**
Hip rotation	PC1	0.32	0.13	0.37	0.082	−0.052	0.81
	PC2	0.013	0.95	0.17	0.43	−0.29	0.18
	PC3	0.41	0.052	−0.0042	0.98	−0.11	0.62

**Fig 7:**
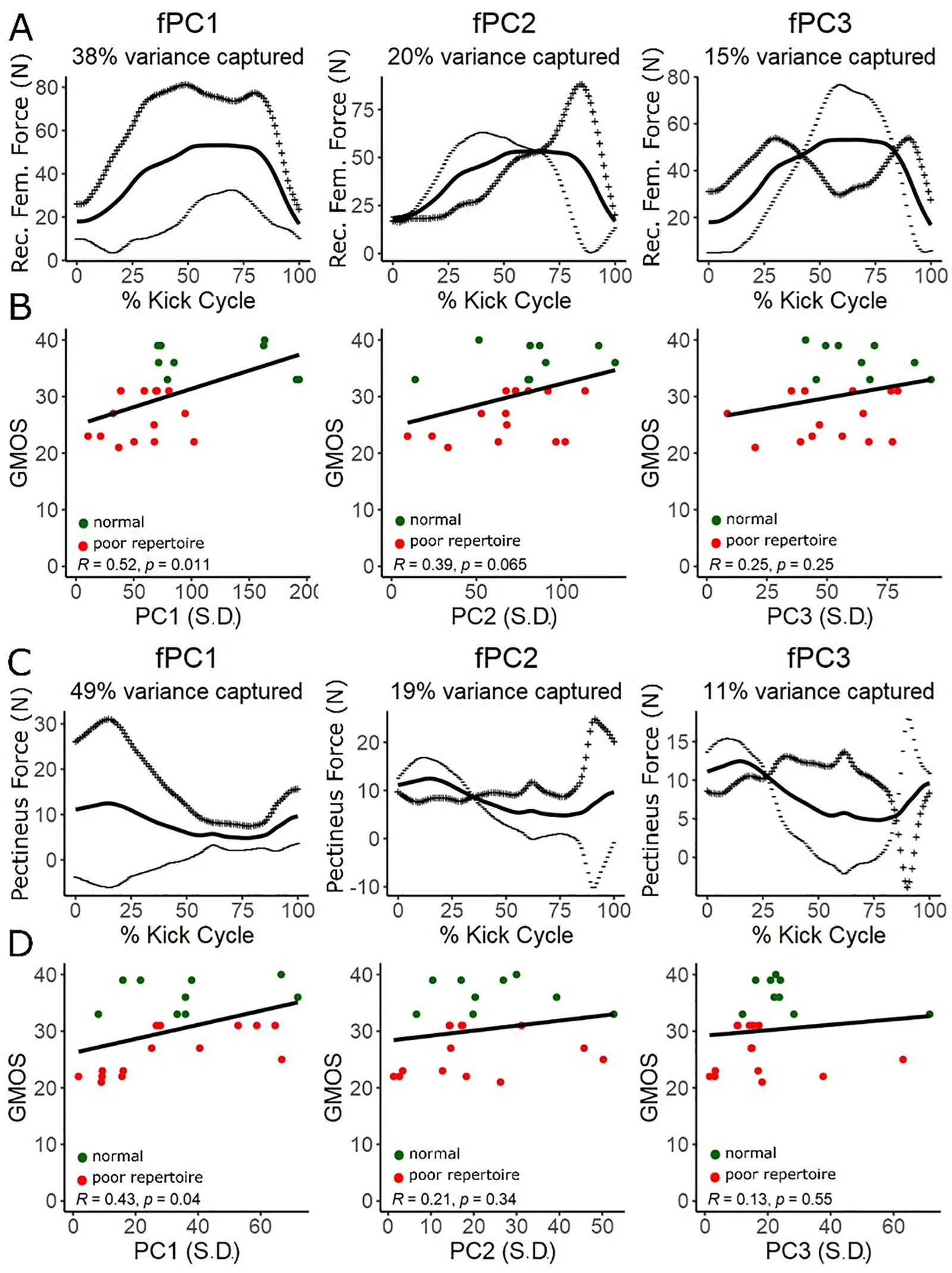
Variation in quadriceps force during kicking is significantly associated (p < 0.05) with GMOS score. A) PC reconstructions of the rectus femoris force curve. PC1: overall rectus femoris force magnitude, PC2: late kick rectus femoris activation, PC3: continuous to bi-phasic rectus femoris activation. B) Pearson’s correlations of the standard deviation (SD) of rectus femoris PC scores for each subject and the GMOS scores. C) PC reconstructions of the pectineus force curve. PC1: early-kick pectineus force magnitude, PC2: late-kick pectineus activation, PC3: positive to negative force bias during mid-late kick. D) Pearson’s correlations of the standard deviation (SD) of pectineus PC scores for each subject and the GMOS scores. Solid line; mean. + line; mean +2 × SD. – line; mean −2 × SD. Red points represent infants assigned a ‘poor repertoire’ of movement score in the assessment. Green points represent infants assigned a ‘normal’ score. PC; principal component. SD; standard deviation. GMOS; general movement optimality score. Lower GMOS score = higher risk of CP. Variance captured; the variation of the original raw data captured by the PC.

**Fig 8:**
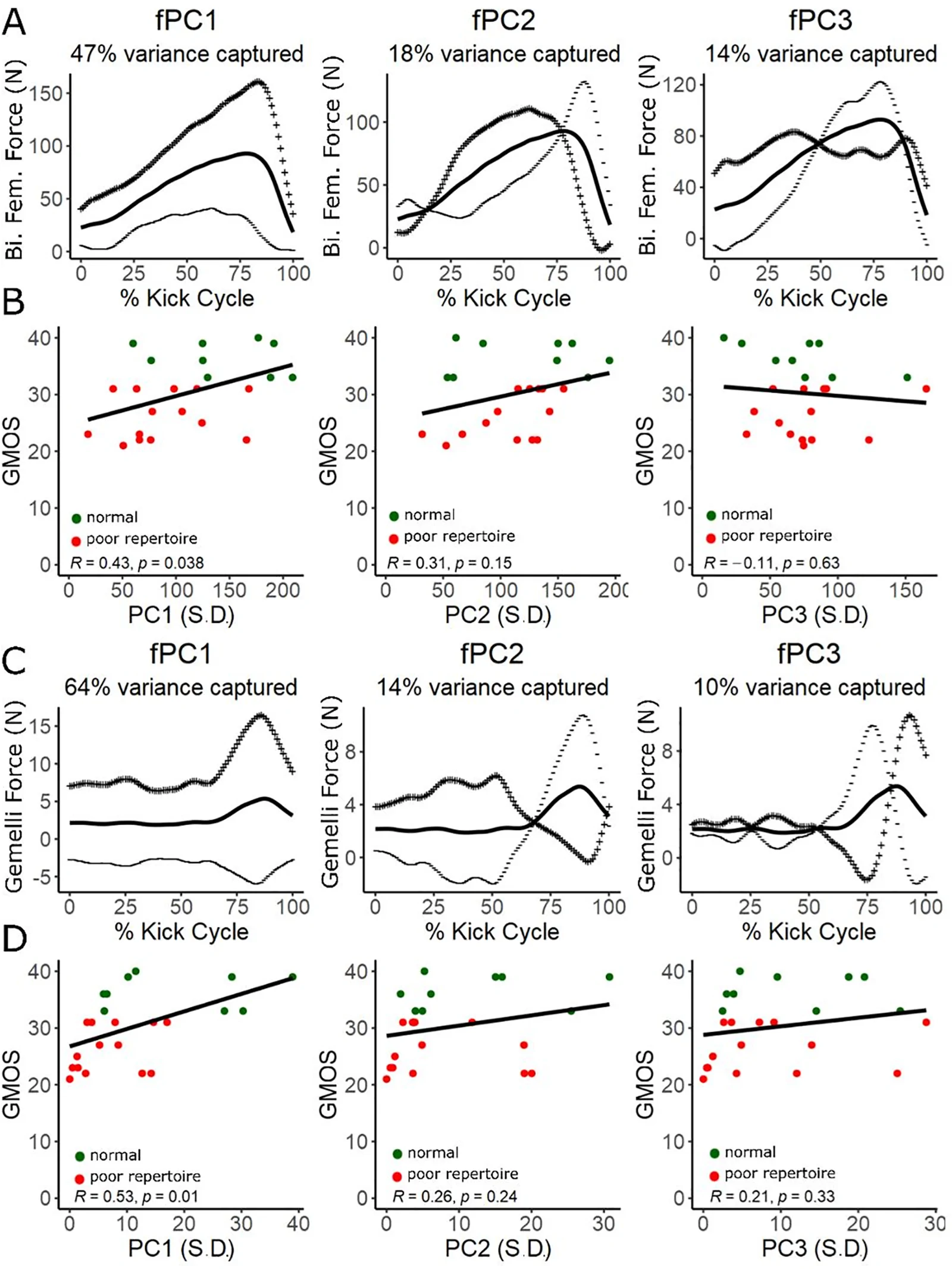
Variation in hamstrings force during kicking is significantly associated (p < 0.05) with GMOS scores. A) PC reconstructions of the biceps femoris force curve. PC1: overall biceps femoris force magnitude, PC2: mid-to-late kick biceps femoris activation, PC3: continuous to bi-phasic biceps femoris activation. B) Pearson’s correlations of the standard deviation (SD) of biceps femoris PC scores for each subject and the GMOS scores. C) PC reconstructions of the gemelli force curve. PC1: late stance gemelli activation force, PC2: mid-to-late kick activation pattern, PC3: late-kick activation pattern. D) Pearson’s correlations of the standard deviation (SD) of gemelli PC scores for each subject and the GMOS scores. Solid line; mean. + line; mean +2 × SD. – line; mean −2 × SD. Red points represent infants assigned a ‘poor repertoire’ of movement score in the assessment. Green points represent infants assigned a ‘normal’ score. PC; principal component. SD; standard deviation. GMOS; general movement optimality score. Lower GMOS score = higher risk of CP. Variance captured; the variation of the original raw data captured by the PC.

For thigh muscle forces, a significant positive association was identified between the captured variation of the overall magnitude of the rectus femoris force curve (PC1, R = 0.52, p < 0.05), early kick pectineus force (PC1, R = 0.43, p < 0.05), and late-kick biceps femoris force (PC1, R = 0.43, p = 0.038) with GMOS scores (Table 2, Figs 7 and 8). At the hip, variation of the late kick gemelli force (PC1, R = 0.53, p = 0.01) significantly correlated with GMOS scores, but there was also a non-significant moderate association with the psoas activation force pattern (PC1, R = 0.42, p = 0.057) (Fig 8, Table 2 and supplementary table 2). Finally, there were no correlations or trends found between variations of calf muscle forces and GMOS score (Supplementary table 2). We conclude that variation of thigh and hip muscle activation forces during kicking are associated with GMOS scores.

**Table 2:** Statistically significant (p < 0.05) Pearson’s correlations between lower limb muscle force principal component standard deviations and the General Movement Optimality Score (GMOS), gestational age at birth and corrected age at assessment.

		GMOS		Gestational Age (at birth)		Corrected Age (at assessment)	
		R	*p*	R	*p*	R	*p*
Rectus Femoris	PC1	**0.52**	**0.011**	0.11	0.630	−0.41	0.053
	PC2	0.39	0.065	0.37	0.079	0.19	0.370
	PC3	0.25	0.250	−0.04	0.870	0.04	0.850
Pectineus	PC1	**0.43**	**0.040**	0.02	0.920	0.03	0.900
	PC2	0.21	0.340	−0.09	0.680	−0.28	0.190
	PC3	0.13	0.550	−0.09	0.700	−0.36	0.091
Biceps Femoris	PC1	**0.43**	**0.038**	0.16	0.460	−0.30	0.170
	PC2	0.31	0.150	0.28	0.200	0.18	0.420
	PC3	−0.11	0.630	0.34	0.110	0.15	0.500
Gemelli	PC1	**0.53**	**0.010**	**0.44**	**0.036**	−0.27	0.220
	PC2	0.26	0.240	0.39	0.066	−0.25	0.260
	PC3	0.21	0.330	**0.50**	**0.016**	−0.07	0.740
Medial Gastroc	PC1	0.06	0.790	**0.42**	**0.044**	0.20	0.350
	PC2	0.09	0.700	0.36	0.092	0.21	0.330
	PC3	−0.05	0.840	**0.69**	**0.000**	0.23	0.300

Variations in hip abduction (PC3, R = 0.49, p < 0.05) and ankle dorsiflexion (PC2, R = 0.53, p = 0.001, and PC3, R = 0.52, p < 0.05) were significantly associated with the gestational age at birth (Table 1). For muscle forces, variations in gemelli force curves (PC1, R = 0.44, p < 0.05 and PC3, R = 0.5, p < 0.05) and medial gastrocnemius force curves (PC1, R = 0.42, p < 0.05, and PC3, R = 0.69, p < 0.001) correlated with gestational age at birth (Table 2). Lower limb kinematic and muscle force variations did not associate strongly with corrected age at examination, except for a moderate trend identified with the variation of hip abduction (PC3, R = 0.4, p = 0.061) (Table 1). In summary, we found that increased variation in kinematics and muscle forces during kicking movements is associated with gestational age at birth, but not corrected age at time of assessment.

### Partial least squares discriminant analysis (PLS-DA)

**Fig 9:**
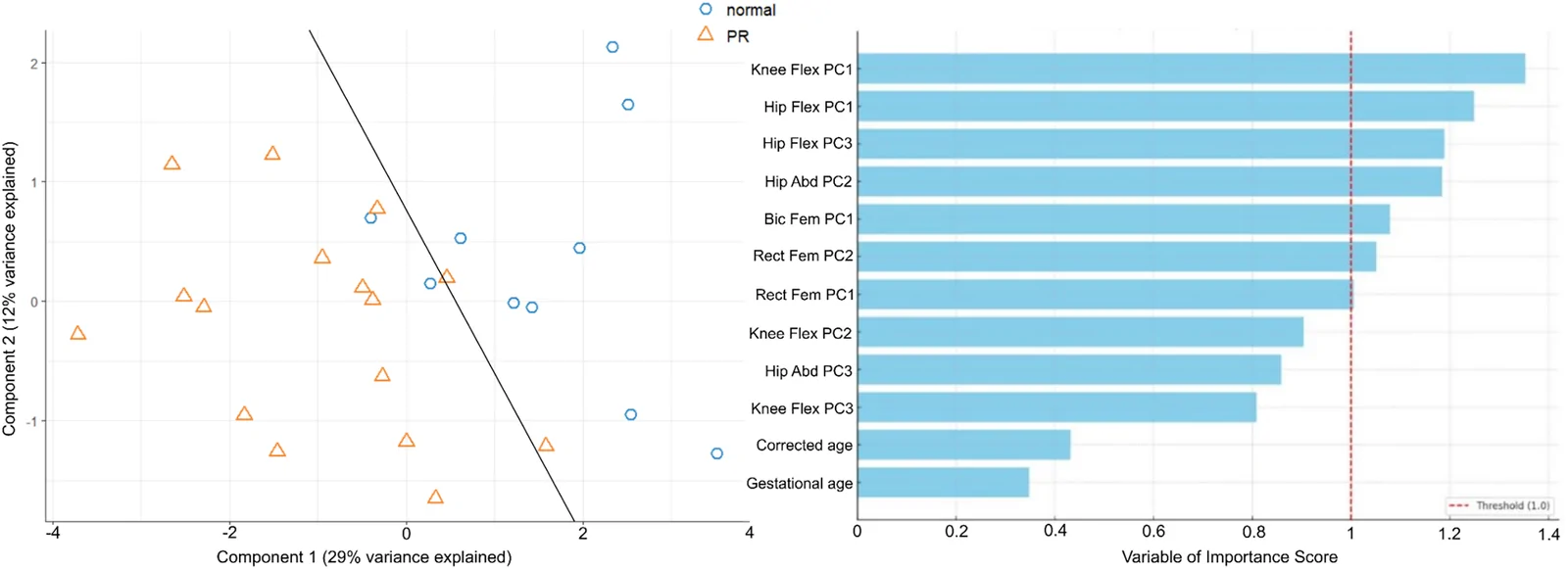
‘Normal’ or ‘poor repertoire’ of movement infants can be predicted with 85% accuracy using objective markers of movement. Partial Least Squares Discriminant Analysis (PLS-DA) of infants with ‘normal’ and ‘poor repertoire’ of movement as assigned in the GMOS. Predictor variables included lower limb kinematic PC scores, lower limb muscle force PC scores, gestational age and corrected age. The model produced a specificity of 90% for predicting ‘poor repertoire’ infants with a sensitivity of 81.25% (A) PLS-DA separation plot indicating a good separation between ‘normal’ and ‘poor repertoire’ infants with 3 out of 26 incorrectly labelled samples. *p*-value = 0.049 from 1000 permutations. (B) Variable Importance in Projection plot showing the most valuable variables in the discrimination of normal and PR infants. Variables scoring >1 have more than average weighting on the components. PLS-DA; partial least squares discriminant analysis. PR; poor repertoire.

The PLS-DA model showed separation between the normal and PR categorised infants and achieved an overall classification accuracy of approximately 85.08% (Fig 9A). The sensitivity and specificity of the model were computed to be 90% and 81.25%, respectively. This indicates the model's ability to correctly identify 90% of the ‘PR’ infants and 81.25% of the ‘normal’ infants. The p-value obtained from the permutation test was approximately 0.049, indicating borderline statistical significance for the model. Extracting the variable weights revealed that the most important variables in projection (more important than average) were PC1 of the knee flexion waveform, PC1 and PC3 of the hip flexion waveform, PC2 of the hip abduction waveform, PC1 of the biceps femoris and PC2 of the rectus femoris (Fig 9B). In summary, the PLS-DA indicates that kinematic features in the lower limbs are the highest contributing predictors of normal and PR of general movements, followed by muscle force curves. Gestational age at birth and corrected age were least predictive of normal vs PR general movements.

## Discussion

This study provides a novel insight into lower limb biomechanical and neuromuscular function surrounding common kicking movements of neonatal infants. We first established a normative range of estimated lower limb kinematics and muscle forces during kicking cycles using a subject-specific musculoskeletal modelling approach. We then found that variations in knee and hip kinematic and thigh and hip muscle force profiles are significantly correlated with GMOS scores, clinically used to identify CP. We also report that variations of hip and ankle kinematics are associated with gestational age at birth. Taken together, these findings corroborate the hypotheses that objective variations in lower limb kinematics and neuromuscular function are reduced in infants with lower GMOS scores and lower gestational age at birth, but not corrected age at assessment. Finally, we showed that kinematic and muscle force variance parameters during kicking could discriminate between infants categorised with ‘normal’ or ‘PR’ general movements, with an accuracy of approximately 85%. In summary, our findings demonstrate that musculoskeletal modelling of neonatal infants may represent a useful approach to evaluating the biomechanical and neuromuscular function of preterm infants of healthy condition or sensory-motor impairment in both continuous severity scales and binary classifications, and holds potential for the development of prediction models of CP development, subject to further validation.

Kinematic variations in hip and knee rotations during kicking movements were correlated with the quality of general movements as scored on the GMOS, and thus the degree of motor development. This result was expected and validates our approach to the quantification of movement variation, since the GMOS primarily considers movement variety in its subjective scoring. However, this study is the first to identify related objective biomechanical measures to the GMOS, which opens opportunities for objective quantification of CP risk aligned with current clinical practice. Previous studies have shown that patterns of in-phase inter-limb and inter-joint rotations of the lower limbs of infants during kicking become progressively decoupled in early life [23,25–31]. However, this increasingly decoupled and varying movement with age is delayed in infants with white matter damage [23,24,31]. Therefore, while previous studies have shown that variation in inter-limb and inter-joint movement are affected by neuromotor impairment, this study demonstrates that intra-joint movements are also potentially affected.

Our study found that decreased variations in hip and thigh muscle forces corresponded with lower GMOS scores. This suggests that infants at a higher risk of developing CP might have a limited range of muscle activation strategies during spontaneous movements. Notably, the force exerted by the biceps femoris, rectus femoris, and gemelli were the most indicative of GMOS scores. We are not aware of previous studies investigating muscle activation force in infants at risk of CP, however it is well established that muscle pathologies such as hypertonia often emerge alongside CP development [37]. Since lower limb musculoskeletal development depends on a normal range of muscle stimulus, the reduced variation of muscle forces in infants at higher risk of CP could be associated with secondary musculoskeletal pathologies. Interestingly, our results identified reduced variation in biceps femoris activation force (one of the muscles of the hamstring) in infants with low GMOS scores, which could be linked to hamstring spasticity commonly found in spastic CP, a debilitating secondary pathology [38]. Previous work has shown that physiotherapy aiming to increase movement variation during infancy can increase the quality of general movements [21,39], therefore biomechanical indicators of movement and muscle activation such as that identified in this study may be well suited to evaluating physiotherapeutic efficacy of such approaches.

Kinematic variation in hip and ankle movement was associated with gestational age at birth, but not corrected age at time of motion analysis. However, the variation of the three parameters were not found to be associated with the GMOS, therefore further analysis with a larger dataset would be needed to further validate this result. The relationships between movement variation and age were not as pronounced as expected, given previous work that has shown that inter-segmental coupling of kicking movement becomes progressively less in-phase over the first two weeks post-birth, and larger differences in inter-segment coupling across the entire limb are seen over the course of six months post-birth [23,26–29]. However, it is notable that the range of age at assessment within this study was narrow, with all infants being within the ‘writhing’ stage of motor development observed in the first 3 months post-birth. A larger study involving infants of a wider range of ages from both the writhing, fidgety (3–5 months) and voluntary (over 5 months) stages of development is warranted for the development of stronger biomechanical indicators of motor maturity.

Our PLS-DA model produced a detection accuracy of ~85% with a sensitivity of 90% and specificity of 81.25%. However, it is notable that the permutation test indicated borderline statistical significance, suggesting that the observed classification performance should be interpreted with caution given the limited sample size and potential model instability. In addition, the dataset was moderately imbalanced (10 ‘normal’ vs 16 ‘PR’ infants), which may bias performance metrics such as accuracy and should be considered when interpreting classification outcomes.

Our classification performance is in the higher range of previous attempts to detect abnormal movements in infants using motion analysis, which achieved sensitivities of 73–90% or detection accuracies of up to 88% [9–18]. Whereas video-based machine learning studies have reported higher classification performance, in some cases exceeding 90% accuracy or AUC values approaching 0.8–0.99, particularly when using deep learning or feature fusion approaches applied to pose estimation data [11,12,14,15,40–42]. A recent systematic review further contextualises these findings, showing that sensor-based approaches typically achieve sensitivities of ~49–85% and specificities of ~57–83%, whereas video-based machine learning approaches more consistently exceed 80% for both sensitivity and specificity, with some studies reporting accuracies above 90% in larger cohorts [43]. Within this landscape, our results are comparable to higher-performing video-based approaches and exceed typical ranges reported for smaller sensor-based datasets. However, it is notable that other studies that achieved higher classification performances assessed infants at later developmental stages, some within the fidgety period, when abnormal movements are more detectable with the GMA compared to the writhing stage [5]. While our accuracy requires further improvement to be of clinical value compared to the GMA which has a reported sensitivity of 98%, it is notable that this sensitivity included infants that displayed ‘cramped synchronised’ and ‘chaotic’ movements that were not included within this study due to low numbers. Several limitations of the study including small sample size and limited per subject data likely limited classification accuracy. It is notable however that our discriminant model correctly classified subjects in the extremes where diagnosis with the GMA is considered with much higher certainty.

Our discriminant analysis also revealed that variations in knee flexion, hip flexion, hip abduction, biceps femoris force and rectus femoris force were most discriminatory between ‘normal’ and ‘PR’ movements. The higher discriminatory power of kinematic variables suggests that, when using a musculoskeletal modelling approach, kinematic measures might be more sensitive than neuromuscular outputs in predicting CP risk. It is notable that modelling muscle force outputs necessitates more assumptions than kinematic outputs, such as muscle insertion points, muscle-tendon force-activation relationships, and the muscle length-tension properties that are inherent to the generic musculoskeletal model. While inverse kinematic outputs derived from generic musculoskeletal models introduces kinematic output inaccuracies, particularly since the model used was developed around adult morphometric parameters, the additional muscle activation assumptions introduce even further inaccuracy into the muscle force data. Our findings regarding muscle activation could therefore benefit from further validation with infant-specific musculoskeletal models yet to be developed, or other more direct methods of measurement such as electromyography.

Our findings suggest that musculoskeletal modelling could, with further validation, serve as a practical adjunct to the GMOS in clinical settings, providing objective measures of lower limb kinematics and muscle forces to inform early intervention strategies. While the integration of current motion capture systems in clinical settings is limited by cost and the need for specialist personnel, advances in markerless pose estimation using simpler and cheaper camera systems and automated musculoskeletal modelling pipelines are making such approaches increasingly feasible. This study offers proof of concept that biomechanical metrics can align with established clinical assessments, though larger-scale validation will be needed to confirm cost-effectiveness, workflow integration, and clinical utility for early cerebral palsy detection and monitoring.

In conclusion, this work revealed that objective variations in lower limb kinematics and muscle forces during kicking movements are associated with GMOS scores, the current gold standard clinical measure of CP risk. The biomechanical indicators identified in this study and that of others may be valuable for investigating the developmental trajectory of infants with diagnosed CP undergoing treatment, or to compare interventions. Furthermore, our approach provides a complementary perspective to CP prediction alongside computer vision methods by improving the translational relevance of the outputs, which potentially provide mechanistic and clinically interpretable kinematic and muscle force markers of abnormal movements. In addition, our findings suggest that alongside currently emerging automated pose estimation algorithms and biomechanical modelling pipelines, it may be possible to build low-cost supportive tools for CP detection and monitoring of motor development. Such tools could be universally accessible in clinic to alleviate cost and pressures associated with maintaining clinical personnel, and provide value in supporting staff recalibration or training methodologies.

## Supporting information

S1 Table All Pearson’s correlations between lower limb kinematic principal component standard deviations and the General Movement Optimality Score (GMOS), gestational age at birth and corrected age at assessment. (DOCX)

S2 Table All Pearson’s correlations between lower limb muscle force principal component standard deviations and the General Movement Optimality Score (GMOS), gestational age at birth and corrected age at assessment. (DOCX)

S3 Table Raw kinematic and kinetic baby kick data. (XLSX)

S4 Table Subject GMOS scores and ages. (XLSX)
