# Supplementary material for: Infant limb kinematics and muscle force variation during kicking is associated with clinical cerebral palsy risk measures
Source: PLoS One. 2026 Sep 8;21(9):e0357214. doi: 10.1371/journal.pone.0357214 (PMC13552763; doi:10.1371/journal.pone.0357214)
Supplement: Supplementary file 1 [file pone.0357214.s001.docx]

**S1 Table** – **All Pearson’s correlations between lower limb kinematic principal component standard deviations and the General Movement Optimality Score (GMOS), gestational age at birth and corrected age at assessment.**

|  |  | GMOS | | Gestational Age (at birth) | | Corrected Age (at assessment) | |
| --- | --- | --- | --- | --- | --- | --- | --- |
|  |  | R | *p* | R | *p* | R | *p* |
| Knee flexion | PC1 | **0.59** | **0.0017** | -0.12 | 0.55 | -0.25 | 0.21 |
|  | PC2 | **0.44** | **0.024** | 0.06 | 0.77 | 0.019 | 0.93 |
|  | PC3 | **0.58** | **0.0018** | 0.31 | 0.12 | 0.16 | 0.43 |
| Ankle flexion | PC1 | 0.38 | 0.075 | 0.27 | 0.22 | 0.11 | 0.6 |
|  | PC2 | 0.38 | 0.071 | **0.53** | **0.0098** | 0.2 | 0.37 |
|  | PC3 | 0.3 | 0.16 | **0.52** | **0.011** | 0.3 | 0.16 |
| Hip flexion | PC1 | 0.37 | 0.079 | -0.044 | 0.84 | -0.17 | 0.43 |
|  | PC2 | -0.073 | 0.74 | 0.015 | 0.94 | -0.14 | 0.53 |
|  | PC3 | **0.44** | **0.036** | -0.3 | 0.16 | -0.077 | 0.73 |
| Hip abduction | PC1 | -0.0061 | 0.98 | 0.037 | 0.87 | -0.28 | 0.19 |
|  | PC2 | 0.39 | 0.062 | 0.056 | 0.8 | -0.14 | 0.51 |
|  | PC3 | **0.48** | **0.022** | **0.49** | **0.017** | 0.2 | 0.261 |
| Hip rotation | PC1 | 0.32 | 0.13 | **0.37** | **0.082** | -0.052 | 0.81 |
|  | PC2 | 0.013 | 0.95 | 0.17 | 0.43 | -0.29 | 0.18 |
|  | PC3 | 0.41 | 0.052 | -0.0042 | 0.98 | -0.11 | 0.62 |
