# Supplementary material for: Infant limb kinematics and muscle force variation during kicking is associated with clinical cerebral palsy risk measures
Source: PLoS One. 2026 Sep 8;21(9):e0357214. doi: 10.1371/journal.pone.0357214 (PMC13552763; doi:10.1371/journal.pone.0357214)
Supplement: Supplementary file 2 [file pone.0357214.s002.docx]

**S2 Table** - **All Pearson’s correlations between lower limb muscle force principal component standard deviations and the General Movement Optimality Score (GMOS), gestational age at birth and corrected age at assessment.**

|  |  | GMOS | | Gestational Age (at birth) | | Corrected Age (at assessment) | |
| --- | --- | --- | --- | --- | --- | --- | --- |
|  |  | R | *p* | R | *p* | R | *p* |
| Gluteus Medius | PC1 | -0.19 | 0.380 | 0.10 | 0.660 | -0.28 | 0.190 |
|  | PC2 | -0.20 | 0.360 | 0.13 | 0.560 | -0.17 | 0.430 |
|  | PC3 | -0.16 | 0.460 | 0.14 | 0.520 | -0.18 | 0.420 |
| Gluteus Maximus | PC1 | 0.09 | 0.680 | 0.10 | 0.640 | -0.02 | 0.940 |
|  | PC2 | -0.26 | 0.230 | -0.05 | 0.820 | 0.18 | 0.420 |
|  | PC3 | 0.01 | 0.960 | 0.14 | 0.520 | 0.27 | 0.220 |
| Iliacus | PC1 | 0.32 | 0.140 | -0.17 | 0.430 | -0.17 | 0.440 |
|  | PC2 | 0.04 | 0.850 | -0.08 | 0.730 | -0.29 | 0.180 |
|  | PC3 | 0.35 | 0.099 | -0.08 | 0.700 | -0.09 | 0.700 |
| Psoas | PC1 | 0.42 | 0.047 | 0.34 | 0.110 | 0.37 | 0.078 |
|  | PC2 | 0.03 | 0.900 | -0.18 | 0.420 | -0.32 | 0.140 |
|  | PC3 | 0.21 | 0.340 | -0.25 | 0.250 | -0.34 | 0.110 |
| TFL | PC1 | -0.36 | 0.091 | 0.06 | 0.800 | 0.02 | 0.930 |
|  | PC2 | -0.41 | 0.054 | 0.13 | 0.560 | -0.05 | 0.820 |
|  | PC3 | -0.17 | 0.440 | -0.17 | 0.440 | -0.16 | 0.470 |
| Piriformis | PC1 | -0.25 | 0.250 | 0.13 | 0.540 | -0.27 | 0.220 |
|  | PC2 | -0.35 | 0.100 | 0.11 | 0.610 | -0.25 | 0.260 |
|  | PC3 | -0.22 | 0.320 | 0.29 | 0.180 | -0.07 | 0.740 |
| Gemelli | PC1 | **0.53** | **0.010** | **0.44** | **0.036** | -0.27 | 0.220 |
|  | PC2 | 0.26 | 0.240 | 0.39 | 0.066 | -0.25 | 0.260 |
|  | PC3 | 0.21 | 0.330 | **0.50** | **0.016** | -0.07 | 0.740 |
| Rectus Femoris | PC1 | **0.52** | **0.011** | 0.11 | 0.630 | -0.41 | 0.053 |
|  | PC2 | 0.39 | 0.065 | 0.37 | 0.079 | 0.19 | 0.370 |
|  | PC3 | 0.25 | 0.250 | -0.04 | 0.870 | 0.04 | 0.850 |
| Vastus Intermedius | PC1 | 0.22 | 0.300 | 0.31 | 0.150 | 0.27 | 0.250 |
|  | PC2 | 0.33 | 0.130 | 0.07 | 0.740 | 0.14 | 0.540 |
|  | PC3 | 0.38 | 0.077 | 0.13 | 0.550 | -0.15 | 0.480 |
| Pectineus | PC1 | **0.43** | **0.040** | 0.02 | 0.920 | 0.03 | 0.900 |
|  | PC2 | 0.21 | 0.340 | -0.09 | 0.680 | -0.28 | 0.190 |
|  | PC3 | 0.13 | 0.550 | -0.09 | 0.700 | -0.36 | 0.091 |
| Sartorius | PC1 | -0.22 | 0.310 | 0.12 | 0.580 | 0.05 | 0.840 |
|  | PC2 | 0.18 | 0.400 | -0.01 | 0.970 | -0.16 | 0.470 |
|  | PC3 | 0.23 | 0.290 | -0.05 | 0.840 | -0.37 | 0.079 |
| Biceps Femoris | PC1 | **0.43** | **0.038** | 0.16 | 0.460 | -0.30 | 0.170 |
|  | PC2 | 0.31 | 0.150 | 0.28 | 0.200 | 0.18 | 0.420 |
|  | PC3 | -0.11 | 0.630 | 0.34 | 0.110 | 0.15 | 0.500 |
| Adductor Magnus | PC1 | 0.03 | 0.900 | -0.07 | 0.760 | -0.28 | 0.200 |
|  | PC2 | -0.14 | 0.520 | 0.19 | 0.380 | -0.02 | 0.920 |
|  | PC3 | -0.09 | 0.670 | 0.26 | 0.230 | -0.24 | 0.270 |
| Gracilis | PC1 | 0.35 | 0.098 | 0.33 | 0.130 | 0.32 | 0.130 |
|  | PC2 | 0.32 | 0.130 | 0.38 | 0.077 | 0.39 | 0.067 |
|  | PC3 | 0.31 | 0.150 | 0.26 | 0.230 | 0.24 | 0.270 |
| Medial Gastroc | PC1 | 0.06 | 0.790 | **0.42** | **0.044** | 0.20 | 0.350 |
|  | PC2 | 0.09 | 0.700 | 0.36 | 0.092 | 0.21 | 0.330 |
|  | PC3 | -0.05 | 0.840 | **0.69** | **0.000** | 0.23 | 0.300 |
| Soleus | PC1 | 0.04 | 0.860 | -0.25 | 0.250 | -0.36 | 0.110 |
|  | PC2 | 0.06 | 0.800 | -0.06 | 0.780 | -0.23 | 0.290 |
|  | PC3 | 0.31 | 0.150 | -0.02 | 0.940 | -0.35 | 0.100 |
| Tibialis Posterior | PC1 | -0.04 | 0.840 | -0.25 | 0.260 | 0.21 | 0.330 |
|  | PC2 | 0.20 | 0.370 | -0.02 | 0.920 | 0.22 | 0.320 |
|  | PC3 | 0.30 | 0.170 | 0.04 | 0.870 | 0.04 | 0.860 |
| Tibialis Anterior | PC1 | -0.13 | 0.560 | -0.24 | 0.225 | -0.37 | 0.081 |
|  | PC2 | 0.14 | 0.510 | -0.30 | 0.159 | -0.22 | 0.032 |
|  | PC3 | -0.08 | 0.710 | -0.36 | 0.091 | -0.37 | 0.087 |
