# Supplementary material for: Infant limb kinematics and muscle force variation during kicking is associated with clinical cerebral palsy risk measures
Source: PLoS One. 2026 Sep 8;21(9):e0357214. doi: 10.1371/journal.pone.0357214 (PMC13552763; doi:10.1371/journal.pone.0357214)
Supplement: Supplementary file 4 [file pone.0357214.s007.docx]

**Response to Editor and Reviewers**

Dear Editorial team and reviewers,

- Thank you for taking the time to review our manuscript and for the valuable feedback
- We have addressed the comments raised by the reviewers and hope that it meets your expectations
- In particular, we have:
  - Made our raw data available publicly
  - Expanded the discussion to satisfy important points raised by the reviewers
  - Tempered the results sections as to not overstate any results
  - Improved the clarity of reporting for certain aspects of the work

**Editor**

When submitting your revision, we need you to address these additional requirements. 1.Please ensure that your manuscript meets PLOS ONE's style requirements, including those for file naming. The PLOS ONE style templates can be found at:

https://journals.plos.org/plosone/s/file?id=wjVg/PLOSOne_formatting_sample_main_body.pdf and

https://journals.plos.org/plosone/s/file?id=ba62/PLOSOne_formatting_sample_title_authors_affiliations.pdf 2.

We note that your Data Availability Statement is currently as follows: All relevant data are within the manuscript and in Supporting Information files. Please confirm at this time whether or not your submission contains all raw data required to replicate the results of your study. Authors must share the “minimal data set” for their submission. PLOS defines the minimal data set to consist of the data required to replicate all study findings reported in the article, as well as related metadata and methods (<https://journals.plos.org/plosone/s/data-availability#loc-minimal-data-set-definition>).

For example, authors should submit the following data:

- The values behind the means, standard deviations and other measures reported;

- The values used to build graphs;

- The points extracted from images for analysis.

Authors do not need to submit their entire data set if only a portion of the data was used in the reported study.

If your submission does not contain these data, please either upload them as Supporting Information files or deposit them to a stable, public repository and provide us with the relevant URLs, DOIs, or accession numbers. For a list of recommended repositories, please see <https://journals.plos.org/plosone/s/recommended-repositories>.

If there are ethical or legal restrictions on sharing a de-identified data set, please explain them in detail (e.g., data contain potentially sensitive information, data are owned by a third-party organization, etc.) and who has imposed them (e.g., an ethics committee). Please also provide contact information for a data access committee, ethics committee, or other institutional body to which data requests may be sent. If data are owned by a third party, please indicate how others may request data access.

3. Please upload a copy of Figure 9, to which you refer in your text on page 18. If the figure is no longer to be included as part of the submission please remove all reference to it within the text.

4. If the reviewer comments include a recommendation to cite specific previously published works, please review and evaluate these publications to determine whether they are relevant and should be cited. There is no requirement to cite these works unless the editor has indicated otherwise.

5. Please review your reference list to ensure that it is complete and correct. If you have cited papers that have been retracted, please include the rationale for doing so in the manuscript text, or remove these references and replace them with relevant current references. Any changes to the reference list should be mentioned in the rebuttal letter that accompanies your revised manuscript. If you need to cite a retracted article, indicate the article’s retracted status in the References list and also include a citation and full reference for the retraction notice.

**Reviewer 1**

*This manuscript demonstrates that the objective biomechanical measures using kinematics and muscle force waveforms can provide quantitative predictors of cerebral palsy risk. The hypothesis is sounded and interesting. However, this work mentioned several limitations that need to further improve for clinical practice. To improve clarity and interest for readers, there are several points needed to clarify and address as follows:*

We thank the reviewer for the positive comments and suggestions to improve the manuscript. We provide the responses to each point below:

*In the abstract, it is better to clearly address the number of infants and the method for motion capture. Furthermore, the quantitative results should be additionally provided.*

The number of infants included in the study has now been explicitly stated (Abstract: line 37), and a brief description of the motion capture methodology has been added (Abstract: line 38) to provide additional context. While we agree that inclusion of quantitative results would further strengthen the abstract, strict journal word limits prevented the addition of further numerical detail without compromising essential background and framing; however, the key quantitative findings are clearly presented in the Results section.

*In the motion capture (Line 184), what type of sensors were used. Please indicate type and model of the sensors.*

Polhemus Liberty electromagnetic wired sensors were used for motion capture, and this has now been explicitly clarified in the manuscript (Methods: lines 185-186).

*As the authors stated (Line 226), “only a single limb was used per infant”, please provide the reasons why only a single limb. Furthermore, which limb that authors chose.*

A single limb per infant was analysed to ensure statistical independence. GMOS scores are a single score assigned at the whole-infant level and thus correlation analysis with both left and right kinematic/force values would have led to pseudo-replication. Given the generally symmetric nature of spontaneous kicking behaviour in early infancy, one limb was considered representative of overall movement patterns and was selected based on the number of recorded kicks and overall data quality when both limbs were available. The methods section has been revised to clarify these points (Methods: lines 227-231).

*In the Results section, please provide more information on hip abduction and PC scores and GMOS scores. Because it shows a significant positive association (R=0.48, p<0.05), including showing as a figure. But the authors showed Hip rotation as in Figure 6C.*

Thank you for pointing this out this error. The inclusion of hip rotation instead of hip abduction in figure 6 was unintentional, as there were no significant associations of GMOS scores with the hip rotation scores. Hip rotation has now been appropriately replaced with Hip Abduction as suggested.

*In the Results section, it talks about the sensitivity and specificity of the PLS-DA model. It is better to describe the method to determine them in the Methods section. This will give a clarity how to determine this PLS-DA model’s performance.*

We have now updated the relevant methods section to explicitly describe how model performance metrics, including sensitivity and specificity, were calculated using the leave-one-out cross-validation approach (lines 276-280).

*In the discussion, it is better if the authors can further discuss the findings with the existing works that use markerless motion captures such as image analysis and machine learning to detect movement disorders of infants, including cerebral palsy detection. Several studies have been conducted markerless motion captures such as:*

*- Wu Q, Xu G, Wei F, Kuang J, Zhang X, Chen L, Zhang S. Automatically measure the quality of infants’ spontaneous movement via videos to predict the risk of cerebral palsy. IEEE Transactions on Instrumentation and Measurement. 2021 Nov 8;70:1-1.*

*- Sakkos D, Mccay KD, Marcroft C, Embleton ND, Chattopadhyay S, Ho ES. Identification of abnormal movements in infants: A deep neural network for body part-based prediction of cerebral palsy. IEEE access. 2021 Jun 29;9:94281-92.*

*- Passmore E, Kwong AL, Greenstein S, Olsen JE, Eeles AL, Cheong JL, Spittle AJ, Ball G. Automated identification of abnormal infant movements from smart phone videos. PLOS digital health. 2024 Feb 22;3(2):e0000432.*

*- Rajpopat S, Kumar S, Punn NS. Cerebral palsy detection from infant using movements of their salient body parts and a feature fusion model. The Journal of Supercomputing. 2025 Jan;81(1):106.*

We have now expanded the discussion to include a more detailed comparison with recent markerless motion capture and video-based machine learning approaches for detecting abnormal infant movements, including the studies suggested. Specifically, we have incorporated our classification performance relative to both earlier sensor-based approaches and more recent video-based machine learning methods. This includes reporting that recent deep learning and feature fusion approaches applied to pose estimation data have achieved higher classification performance in some settings, while also noting important differences in dataset size, population, and outcome definitions. We also clarified that our study differs in its primary objective, in that our approach using biomechanical variables provides mechanistic insight into kinematics and muscle forces aligned with GMOS scoring and clinical interpretation. These additions can be found in the revised Discussion (Discussion: lines 525–536, and 580-584).

*Figure 9A, there are some marks (x) out of the frame ( 2 red marks and 1 blue mark are not fully shown on the plot.*

Thank you for pointing out this error in the plot. The PLS-DA figure 9A has been reworked to include all points clearly within the plot boundaries.

**Reviewer 2**

*The manuscript presents a well-designed study where authors expand the application of motion-capture technology to characterize early-term infant movement, specifically focusing on variability of the movements (kicks). It explores using a continuous score as well as more traditional approach of classification of normal versus poor repertoire, as well as nvestigation of how variability of movements relates to gestational and corrected age. I’d like to commend the authors for extending their analysis to younger infants, as many existing studies focus on older ages. fPCA is a good metric to work with movements to capture variability.*

We thank the reviewer for the positive comments and suggestions to improve the manuscript. We provide the responses to each point below:

*Some recommendations that authors might consider to improve the overall quality of the manuscript:*

*Overall content:*

*The literature overview in the introduction and discussion does not account for past two years. Given developments in the field since then (e.g., some of the works presented in the following systematic review - https://doi.org/10.1038/s41746-025-01450-3 ), authors may wish to incorporate more recent studies to ensure the framing and discussion reflect the current state of knowledge. While it is not critical for the validity of the results, it would strengthen the overall manuscript and put in it context of modern trends in the field.*

We have updated the text to better reflect current advances in automated detection of abnormal infant movements. Specifically, we have incorporated recent video-based machine learning studies (e.g., Passmore et al., 2024; Rajpopat et al., 2025) and included discussion of the suggested systematic review (Deng et al., 2025), which provides a broader overview of performance across sensor-based and video-based approaches. These studies help to clarify that while video-based approaches often report higher classification performance, our results are comparable to the higher range of previously reported methods. These revisions can be found in the discussion (Discussion: lines 525-536).

*On the modern trends – the health policy piece seem to be leaning toward video-based assessment and GMA score (recent collaborative effort DOI: 10.1016/j.eclinm.2025.103379 ) - authors might consider to highlighting specific advantages of using motion capture vs video, e.g., as more precise research tool etc.*

We have clarified that our study provides a complementary perspective by focusing on biomechanical and neuromuscular variability as predictors of GMOS, rather than relying solely on features from video data. This addition improves the translational relevance of the manuscript by distinguishing between approaches optimised for classification accuracy and those, such as ours, that aim to provide mechanistic and clinically interpretable biomarkers (Discussion: lines 580-584).

*It is mentioned that using adult model could introduce inaccuracy into muscle activation assessment, however same could be argued for kinematics, albeit to a smaller degree, in particular for parameters like hip flexion. Authors might consider strengthen the limitation statement.*

Having reviewed the wording we agree that the inaccuracies of muscle activation was implied in our modelling approach, but not kinematics. This statement has now been edited to better highlight this limitation (Discussion: lines 555-558).

*Reporting:*

*Considering that the PLS-DA (doi: 10.1186/s12859-019-3310-7) model is boarder-line statistically significant, dataset is small and somewhat unbalanced, broad claims cannot be made with full confidence. And while authors do mention in the limitations the small dataset size, some statements in the discussion are quite strong. Additionally, reporting further metrics more to account for somewhat unbalanced dataset might be beneficial.*

We have revised the manuscript to ensure that all statements regarding the discriminant analysis and its implications are appropriately moderated. Specifically, we have softened language throughout the results and discussion text with more cautious phrasing (please see tracked changes). We have also reduced the strength of claims relating to clinical applicability and future predictive modelling. In addition, we have expanded the discussion of limitations to more explicitly acknowledge the borderline permutation test result on the PLS-DA and unbalanced group dataset numbers (Discussion: lines 515-520).

*Data statement does not match the materials included – data necessary to replicate their study’s findings is not included in the supplementary materials.*

This raw data is now included as supporting information table S3 and S4.
