# Supplementary material for: Infant limb kinematics and muscle force variation during kicking is associated with clinical cerebral palsy risk measures
Source: PLoS One. 2026 Sep 8;21(9):e0357214. doi: 10.1371/journal.pone.0357214 (PMC13552763; doi:10.1371/journal.pone.0357214)
Supplement: Peer review history

# Peer review: Decision Letter 0

**DOI:** 10.1371/journal.pone.0357214.r001

**Authors:** Dimitrios Sokratis Komaris

26 Nov 2025

Dear Dr. Khatib,

Thank you for submitting your manuscript to PLOS ONE. After careful consideration, we feel that it has merit but does not fully meet PLOS ONE’s publication criteria as it currently stands. Therefore, we invite you to submit a revised version of the manuscript that addresses the points raised during the review process.

Please submit your revised manuscript by Jan 10 2026 11:59PM. If you will need more time than this to complete your revisions, please reply to this message or contact the journal office at plosone@plos.org. When you're ready to submit your revision, log on to https://www.editorialmanager.com/pone/ and select the 'Submissions Needing Revision' folder to locate your manuscript file.

- A rebuttal letter that responds to each point raised by the academic editor and reviewer(s). You should upload this letter as a separate file labeled 'Response to Reviewers'.
- A marked-up copy of your manuscript that highlights changes made to the original version. You should upload this as a separate file labeled 'Revised Manuscript with Track Changes'.
- An unmarked version of your revised paper without tracked changes. You should upload this as a separate file labeled 'Manuscript'.

If applicable, we recommend that you deposit your laboratory protocols in protocols.io to enhance the reproducibility of your results. Protocols.io assigns your protocol its own identifier (DOI) so that it can be cited independently in the future. For instructions see: https://journals.plos.org/plosone/s/submission-guidelines#loc-laboratory-protocols. Additionally, PLOS ONE offers an option for publishing peer-reviewed Lab Protocol articles, which describe protocols hosted on protocols.io. Read more information on sharing protocols at https://plos.org/protocols?utm_medium=editorial-email&utm_source=authorletters&utm_campaign=protocols.

We look forward to receiving your revised manuscript.

Kind regards,

Dimitrios Sokratis Komaris, Ph.D

Academic Editor

PLOS ONE

Journal Requirements:

When submitting your revision, we need you to address these additional requirements.

1.Please ensure that your manuscript meets PLOS ONE's style requirements, including those for file naming. The PLOS ONE style templates can be found at

https://journals.plos.org/plosone/s/file?id=wjVg/PLOSOne_formatting_sample_main_body.pdf and

https://journals.plos.org/plosone/s/file?id=ba62/PLOSOne_formatting_sample_title_authors_affiliations.pdf

2. We note that your Data Availability Statement is currently as follows: All relevant data are within the manuscript and in Supporting Information files.

Please confirm at this time whether or not your submission contains all raw data required to replicate the results of your study. Authors must share the “minimal data set” for their submission. PLOS defines the minimal data set to consist of the data required to replicate all study findings reported in the article, as well as related metadata and methods (https://journals.plos.org/plosone/s/data-availability#loc-minimal-data-set-definition).

For example, authors should submit the following data:

- The values behind the means, standard deviations and other measures reported;

- The values used to build graphs;

- The points extracted from images for analysis.

Authors do not need to submit their entire data set if only a portion of the data was used in the reported study.

If your submission does not contain these data, please either upload them as Supporting Information files or deposit them to a stable, public repository and provide us with the relevant URLs, DOIs, or accession numbers. For a list of recommended repositories, please see https://journals.plos.org/plosone/s/recommended-repositories.

If there are ethical or legal restrictions on sharing a de-identified data set, please explain them in detail (e.g., data contain potentially sensitive information, data are owned by a third-party organization, etc.) and who has imposed them (e.g., an ethics committee). Please also provide contact information for a data access committee, ethics committee, or other institutional body to which data requests may be sent. If data are owned by a third party, please indicate how others may request data access.

3. Please upload a copy of Figure 9, to which you refer in your text on page 18. If the figure is no longer to be included as part of the submission please remove all reference to it within the text.

4. If the reviewer comments include a recommendation to cite specific previously published works, please review and evaluate these publications to determine whether they are relevant and should be cited. There is no requirement to cite these works unless the editor has indicated otherwise.

5. Please review your reference list to ensure that it is complete and correct. If you have cited papers that have been retracted, please include the rationale for doing so in the manuscript text, or remove these references and replace them with relevant current references. Any changes to the reference list should be mentioned in the rebuttal letter that accompanies your revised manuscript. If you need to cite a retracted article, indicate the article’s retracted status in the References list and also include a citation and full reference for the retraction notice.

[Note: HTML markup is below. Please do not edit.]

Reviewers' comments:

Reviewer's Responses to Questions

**Comments to the Author**

1. Is the manuscript technically sound, and do the data support the conclusions?

Reviewer #1: Yes

Reviewer #2: Yes

**********

2. Has the statistical analysis been performed appropriately and rigorously?

Reviewer #1: Yes

Reviewer #2: Yes

**********

3. Have the authors made all data underlying the findings in their manuscript fully available?

The PLOS Data policy

Reviewer #1: Yes

Reviewer #2: No

**********

4. Is the manuscript presented in an intelligible fashion and written in standard English?

Reviewer #1: Yes

Reviewer #2: Yes

**********

Reviewer #1: This manuscript demonstrates that the objective biomechanical measures using kinematics and muscle force waveforms can provide quantitative predictors of cerebral palsy risk. The hypothesis is sounded and interesting. However, this work mentioned several limitations that need to further improve for clinical practice.

To improve clarity and interest for readers, there are several points needed to clarify and address as follows:

1. In the abstract, it is better to clearly address the number of infants and the method for motion capture. Furthermore, the quantitative results should be additionally provided.

2. In the motion capture (Line 184), what type of sensors were used. Please indicate type and model of the sensors.

3. As the authors stated (Line 226), “only a single limb was used per infant”, please provide the reasons why only a single limb. Furthermore, which limb that authors chose.

4. In the Results section, please provide more information on hip abduction and PC scores and GMOS scores. Because it shows a significant positive association (R=0.48, p<0.05), including showing as a figure. But the authors showed Hip rotation as in Figure 6C.

5. In the Results section, it talks about the sensitivity and specificity of the PLS-DA model. It is better to describe the method to determine them in the Methods section. This will give a clarity how to determine this PLS-DA model’s performance.

6. In the discussion, it is better if the authors can further discuss the findings with the existing works that use markerless motion captures such as image analysis and machine learning to detect movement disorders of infants, including cerebral palsy detection. Several studies have been conducted markerless motion captures such as

- Wu Q, Xu G, Wei F, Kuang J, Zhang X, Chen L, Zhang S. Automatically measure the quality of infants’ spontaneous movement via videos to predict the risk of cerebral palsy. IEEE Transactions on Instrumentation and Measurement. 2021 Nov 8;70:1-1.

- Sakkos D, Mccay KD, Marcroft C, Embleton ND, Chattopadhyay S, Ho ES. Identification of abnormal movements in infants: A deep neural network for body part-based prediction of cerebral palsy. IEEE access. 2021 Jun 29;9:94281-92.

- Passmore E, Kwong AL, Greenstein S, Olsen JE, Eeles AL, Cheong JL, Spittle AJ, Ball G. Automated identification of abnormal infant movements from smart phone videos. PLOS digital health. 2024 Feb 22;3(2):e0000432.

- Rajpopat S, Kumar S, Punn NS. Cerebral palsy detection from infant using movements of their salient body parts and a feature fusion model. The Journal of Supercomputing. 2025 Jan;81(1):106.

7. Figure 9A, there are some marks (x) out of the frame ( 2 red marks and 1 blue mark are not fully shown on the plot.

Reviewer #2: The manuscript presents a well-designed study where authors expand the application of motion-capture technology to characterize early-term infant movement, specifically focusing on variability of the movements (kicks). It explores using a continuous score as well as more traditional approach of classification of normal versus poor repertoire, as well as nvestigation of how variability of movements relates to gestational and corrected age. I’d like to commend the authors for extending their analysis to younger infants, as many existing studies focus on older ages. fPCA is a good metric to work with movements to capture variability.

Some recommendations that authors might consider to improve the overall quality of the manuscript:

Overall content:

The literature overview in the introduction and discussion does not account for past two years. Given developments in the field since then (e.g., some of the works presented in the following systematic review - https://doi.org/10.1038/s41746-025-01450-3 ), authors may wish to incorporate more recent studies to ensure the framing and discussion reflect the current state of knowledge. While it is not critical for the validity of the results, it would strengthen the overall manuscript and put in it context of modern trends in the field.

On the modern trends – the health policy piece seem to be leaning toward video-based assessment and GMA score (recent collaborative effort DOI: 10.1016/j.eclinm.2025.103379 ) - authors might consider to highlighting specific advantages of using motion capture vs video, e.g., as more precise research tool etc.

It is mentioned that using adult model could introduce inaccuracy into muscle activation assessment, however same could be argued for kinematics, albeit to a smaller degree, in particular for parameters like hip flexion. Authors might consider strengthen the limitation statement.

Reporting :

Considering that the PLS-DA (doi: 10.1186/s12859-019-3310-7) model is boarder-line statistically significant, dataset is small and somewhat unbalanced, broad claims cannot be made with full confidence. And while authors do mention in the limitations the small dataset size, some statements in the discussion are quite strong. Additionally, reporting further metrics more to account for somewhat unbalanced dataset might be beneficial.

It would add clarity to interpretation if authors to include CI for correlations in the supplementary materials.

Data statement does not match the materials included – data necessary to replicate their study’s findings is not included in the supplementary materials.

**********

what does this mean?). If published, this will include your full peer review and any attached files.

If you choose “no”, your identity will remain anonymous but your review may still be made public.

**Do you want your identity to be public for this peer review?**  For information about this choice, including consent withdrawal, please see our Privacy Policy

Reviewer #1: No

Reviewer #2: No

**********

[NOTE: If reviewer comments were submitted as an attachment file, they will be attached to this email and accessible via the submission site. Please log into your account, locate the manuscript record, and check for the action link "View Attachments". If this link does not appear, there are no attachment files.]

To ensure your figures meet our technical requirements, please review our figure guidelines: https://journals.plos.org/plosone/s/figures

You may also use PLOS’s free figure tool, NAAS, to help you prepare publication quality figures: https://journals.plos.org/plosone/s/figures#loc-tools-for-figure-preparation.

NAAS will assess whether your figures meet our technical requirements by comparing each figure against our figure specifications.

# Author comment: Author response to Decision Letter 1

**DOI:** 10.1371/journal.pone.0357214.r002

2 Jul 2026

Response to reviewers uploaded as a word document

Attachment Submitted filename: Response to Editor and Reviewers.docx

# Peer review: Decision Letter 1

**DOI:** 10.1371/journal.pone.0357214.r003

**Authors:** Dimitrios Sokratis Komaris

15 Jul 2026

Dear Dr. Khatib,

Thank you for submitting your manuscript to PLOS ONE. After careful consideration, we feel that it has merit but does not fully meet PLOS ONE’s publication criteria as it currently stands. Therefore, we invite you to submit a revised version of the manuscript that addresses the points raised during the review process.

=========================================

Thank you for submitting your revised manuscript.

I am returning your submission for one additional minor revision. Reviewer 2 has accepted the revised version; however, Reviewer 1 has raised a remaining concern regarding one of the figures. Specifically, the reviewer noted that the plot in the revised manuscript differs from the one presented in the original submission, with the slope of the fitted line appearing to have changed. I understand that the figure may have been reworked during the revision process, but I would appreciate it if you could address the reviewer's comments below and clarify the reason for this difference.

Please revise the manuscript accordingly and resubmit it at your earliest convenience.

Thank you for your cooperation.

=========================================

Please submit your revised manuscript by Aug 29 2026 11:59PM. If you will need more time than this to complete your revisions, please reply to this message or contact the journal office at plosone@plos.org. When you're ready to submit your revision, log on to https://www.editorialmanager.com/pone/ and select the 'Submissions Needing Revision' folder to locate your manuscript file.

- A letter that responds to each point raised by the academic editor and reviewer(s). You should upload this letter as a separate file labeled 'Response to Reviewers'.
- A marked-up copy of your manuscript that highlights changes made to the original version. You should upload this as a separate file labeled 'Revised Manuscript with Track Changes'.
- An unmarked version of your revised paper without tracked changes. You should upload this as a separate file labeled 'Manuscript'.

If you would like to make changes to your financial disclosure, please include your updated statement in your cover letter. Guidelines for resubmitting your figure files are available below the reviewer comments at the end of this letter.

If applicable, we recommend that you deposit your laboratory protocols in protocols.io to enhance the reproducibility of your results. Protocols.io assigns your protocol its own identifier (DOI) so that it can be cited independently in the future. For instructions see: https://journals.plos.org/plosone/s/submission-guidelines#loc-laboratory-protocols. Additionally, PLOS ONE offers an option for publishing peer-reviewed Lab Protocol articles, which describe protocols hosted on protocols.io. Read more information on sharing protocols at https://plos.org/protocols?utm_medium=editorial-email&utm_source=authorletters&utm_campaign=protocols.

As the corresponding author, your ORCID iD is verified in the submission system and will appear in the published article. PLOS supports the use of ORCID, and we encourage all coauthors to register for an ORCID iD and use it as well. Please encourage your coauthors to verify their ORCID iD within the submission system before final acceptance, as unverified ORCID iDs will not appear in the published article. *Only*  the individual author can complete the verification step; PLOS staff *cannot*  verify ORCID iDs on behalf of authors.

We look forward to receiving your revised manuscript.

Kind regards,

Dimitrios Sokratis Komaris, Ph.D

Academic Editor

PLOS One

**Journal Requirements:**

1. If the reviewer comments include a recommendation to cite specific previously published works, please review and evaluate these publications to determine whether they are relevant and should be cited. There is no requirement to cite these works unless the editor has indicated otherwise.

2. Please review your reference list to ensure that it is complete and correct. If you have cited papers that have been retracted, please include the rationale for doing so in the manuscript text, or remove these references and replace them with relevant current references. Any changes to the reference list should be mentioned in the rebuttal letter that accompanies your revised manuscript. If you need to cite a retracted article, indicate the article’s retracted status in the References list and also include a citation and full reference for the retraction notice.

[Note: HTML markup is below. Please do not edit.]

Reviewers' comments:

Reviewer's Responses to Questions

**Comments to the Author**

Reviewer #1: (No Response)

Reviewer #2: All comments have been addressed

**********

2. Is the manuscript technically sound, and do the data support the conclusions?

Reviewer #1: Yes

Reviewer #2: Yes

**********

3. Has the statistical analysis been performed appropriately and rigorously?

Reviewer #1: Yes

Reviewer #2: Yes

**********

4. Have the authors made all data underlying the findings in their manuscript fully available?

The PLOS Data policy

Reviewer #1: Yes

Reviewer #2: Yes

**********

5. Is the manuscript presented in an intelligible fashion and written in standard English?

Reviewer #1: Yes

Reviewer #2: Yes

**********

Reviewer #1: As the authors' responses, several comments have been clearly clarified. However, there are still some comments that the authors have not achieved satisfactory.

1. In the abstract, it is better to rewrite to provide significant quantitative rsults instead of mentioning "strict journal word limits prevented the addition of further numerical detail without compromising essential background and framing". Furthermore, please replace 26 with Twenty six because it is a begining of sentence. Furthermore, it is not necessary to put the company name of instrument in the abstract-Polhemus Liberty .

2. Figure 9A, why the plot in the revision is different from the first version-the slope of the line is different. I understand the authors reworked it. Are there any changes between x-axis and y-axis parameters?

Reviewer #2: I'd like tto thabk the authors for sufficiently addressing the comments and making dull data available.

**********

what does this mean?). If published, this will include your full peer review and any attached files.

If you choose “no”, your identity will remain anonymous but your review may still be made public.

**Do you want your identity to be public for this peer review?**  For information about this choice, including consent withdrawal, please see our Privacy Policy

Reviewer #1: No

Reviewer #2: No

**********

[NOTE: If reviewer comments were submitted as an attachment file, they will be attached to this email and accessible via the submission site. Please log into your account, locate the manuscript record, and check for the action link "View Attachments". If this link does not appear, there are no attachment files.]

To ensure your figures meet our technical requirements, please review our figure guidelines: https://journals.plos.org/plosone/s/figures

You may also use PLOS’s free figure tool, NAAS, to help you prepare publication quality figures: https://journals.plos.org/plosone/s/figures#loc-tools-for-figure-preparation.

NAAS will assess whether your figures meet our technical requirements by comparing each figure against our figure specifications.

# Author comment: Author response to Decision Letter 2

**DOI:** 10.1371/journal.pone.0357214.r004

24 Jul 2026

Dear Editorial team and Reviewers,

Thank you for the update regarding reviewer feedback. Regarding your request to address the reviewers comments regarding the altered slope of the fitted line Figure 9A:

The difference in the slope between the original and repeated PLS-DA score plots results from a reversal in the sign of the latent components. The orientation of a PLS latent variable is mathematically indeterminate - a component and its sign-reversed equivalent describe the same axis through the data. Consequently, reversing the scores and corresponding weights/loadings mirrors the visualisation but does not alter the relative positions of samples, fitted values, explained variance or classification performance. So to summarise, the mirrored presentation represents an arbitrary visual change in component orientation rather than a change in the underlying result. The actual reason the latent component signs were reversed is likely just related to replotting the scores using an updated version of the statistical package in R that was used to improve the aesthetics of the plot. We have double checked the model outputs just to be sure, and they are all exactly the same as that previously reported.

We hope that this addresses the concern from the reviewer and look forward to hearing your recommendation for the manuscript.

Attachment Submitted filename: Response_to_Editor_and_Reviewers_auresp_2.docx

# Peer review: Decision Letter 2

**DOI:** 10.1371/journal.pone.0357214.r005

**Authors:** Valentina Graci

13 Aug 2026

Infant limb kinematics and muscle force variation during kicking is associated with clinical cerebral palsy risk measures

PONE-D-25-42605R2

Dear Dr. Khatib,

We’re pleased to inform you that your manuscript has been judged scientifically suitable for publication and will be formally accepted for publication once it meets all outstanding technical requirements.

Within one week, you’ll receive an e-mail detailing the required amendments. When these have been addressed, you’ll receive a formal acceptance letter and your manuscript will be scheduled for publication.

An invoice will be generated when your article is formally accepted. Please note, if your institution has a publishing partnership with PLOS and your article meets the relevant criteria, all or part of your publication costs will be covered. Please make sure your user information is up-to-date by logging into Editorial Manager at Editorial Manager® and clicking the ‘Update My Information' link at the top of the page. For questions related to billing, please contact billing support.

If your institution or institutions have a press office, please notify them about your upcoming paper to help maximize its impact. If they’ll be preparing press materials, please inform our press team as soon as possible -- no later than 48 hours after receiving the formal acceptance. Your manuscript will remain under strict press embargo until 2 pm Eastern Time on the date of publication. For more information, please contact onepress@plos.org.

Kind regards,

Valentina Graci, PhD

Academic Editor

PLOS One

Additional Editor Comments (optional):

Reviewers' comments:

Reviewer's Responses to Questions

**Comments to the Author**

Reviewer #1: All comments have been addressed

**********

2. Is the manuscript technically sound, and do the data support the conclusions?

Reviewer #1: Yes

**********

3. Has the statistical analysis been performed appropriately and rigorously?

Reviewer #1: Yes

**********

4. Have the authors made all data underlying the findings in their manuscript fully available?

The PLOS Data policy

Reviewer #1: Yes

**********

5. Is the manuscript presented in an intelligible fashion and written in standard English?

Reviewer #1: Yes

**********

Reviewer #1: The authors addressed the reason of the plot of Figure 9A. It is understandable and accepted. This manuscript can be accepted to publish.

**********

what does this mean?). If published, this will include your full peer review and any attached files.

If you choose “no”, your identity will remain anonymous but your review may still be made public.

**Do you want your identity to be public for this peer review?**  For information about this choice, including consent withdrawal, please see our Privacy Policy

Reviewer #1: No

**********

# Editor report: Acceptance letter

**DOI:** 10.1371/journal.pone.0357214.r006

**Authors:** Valentina Graci

PONE-D-25-42605R2

PLOS One

Dear Dr. Khatib,

I'm pleased to inform you that your manuscript has been deemed suitable for publication in PLOS One. Congratulations! Your manuscript is now being handed over to our production team.

At this stage, our production department will prepare your paper for publication. This includes ensuring the following:

* All references, tables, and figures are properly cited

* All relevant supporting information is included in the manuscript submission,

* There are no issues that prevent the paper from being properly typeset

You will receive further instructions from the production team, including instructions on how to review your proof when it is ready. Please keep in mind that we are working through a large volume of accepted articles, so please give us a few days to review your paper and let you know the next and final steps.

Lastly, if your institution or institutions have a press office, please let them know about your upcoming paper now to help maximize its impact. If they'll be preparing press materials, please inform our press team within the next 48 hours. Your manuscript will remain under strict press embargo until 2 pm Eastern Time on the date of publication. For more information, please contact onepress@plos.org.

You will receive an invoice from PLOS for your publication fee after your manuscript has reached the completed accept phase. If you receive an email requesting payment before acceptance or for any other service, this may be a phishing scheme. Learn how to identify phishing emails and protect your accounts at https://explore.plos.org/phishing.

If we can help with anything else, please email us at customercare@plos.org.

Thank you for submitting your work to PLOS One and supporting open access.

Kind regards,

PLOS One Editorial Office Staff

on behalf of

Dr. Valentina Graci

Academic Editor

PLOS One
